# Autopolyploidization affects transcript patterns and gene targeting frequencies in Physcomitrella

**DOI:** 10.1007/s00299-021-02794-2

**Published:** 2021-10-12

**Authors:** Christine Rempfer, Gertrud Wiedemann, Gabriele Schween, Klaus L. Kerres, Jan M. Lucht, Ralf Horres, Eva L. Decker, Ralf Reski

**Affiliations:** 1 Plant Biotechnology, Faculty of Biology, University of Freiburg, Schaenzlestr. 1, 79104 Freiburg, Germany; 2 Spemann Graduate School of Biology and Medicine (SGBM), University of Freiburg, 79104 Freiburg, Germany; 3 GenXPro GmbH, Altenhöferallee 3, 60438 Frankfurt am Main, Germany; 4 Signalling Research Centres BIOSS and CIBSS, Schaenzlestr. 18, 79104 Freiburg, Germany; 5 Present Address: Department of Hematology and Central Hematology Laboratory, Inselspital, Bern University Hospital, University of Bern, 3010 Bern, Switzerland; 6 Present Address: Corteva Agriscience, Pioneer Hi-Bred Northern Europe, Münstertäler Strasse 26, 79427 Eschbach, Germany; 7 Present Address: Scienceindustries, Nordstrasse 15, 8006 Zurich, Switzerland

**Keywords:** DNA repair, Gene targeting, Moss, Physcomitrium, Protoplast regeneration, Whole genome duplication

## Abstract

**Key message:**

**In Physcomitrella, whole-genome duplications affected the expression of about 3.7% of the protein-encoding genes, some of them relevant for DNA repair, resulting in a massively reduced gene-targeting frequency.**

**Abstract:**

Qualitative changes in gene expression after an autopolyploidization event, a pure duplication of the whole genome (WGD), might be relevant for a different regulation of molecular mechanisms between angiosperms growing in a life cycle with a dominant diploid sporophytic stage and the haploid-dominant mosses. Whereas angiosperms repair DNA double-strand breaks (DSB) preferentially via non-homologous end joining (NHEJ), in the moss Physcomitrella homologous recombination (HR) is the main DNA–DSB repair pathway. HR facilitates the precise integration of foreign DNA into the genome via gene targeting (GT). Here, we studied the influence of ploidy on gene expression patterns and GT efficiency in Physcomitrella using haploid plants and autodiploid plants, generated via an artificial WGD. Single cells (protoplasts) were transfected with a GT construct and material from different time-points after transfection was analysed by microarrays and SuperSAGE sequencing. In the SuperSAGE data, we detected 3.7% of the Physcomitrella genes as differentially expressed in response to the WGD event. Among the differentially expressed genes involved in DNA–DSB repair was an upregulated gene encoding the X-ray repair cross-complementing protein 4 (XRCC4), a key player in NHEJ. Analysing the GT efficiency, we observed that autodiploid plants were significantly GT suppressed (*p* < 0.001) attaining only one third of the expected GT rates. Hence, an alteration of global transcript patterns, including genes related to DNA repair, in autodiploid Physcomitrella plants correlated with a drastic suppression of HR.

**Supplementary Information:**

The online version contains supplementary material available at 10.1007/s00299-021-02794-2.

## Introduction

The duplication of entire genomes leads to polyploidy and occurs in many cell types and organisms. The resulting polyploids often differ from their progenitors, and are mostly viewed as aberrant or not successful in evolutionary terms. In contrast, evidence is accumulating that polyploidization may be a driving force in evolution as it increases the adaptive potential in stressful conditions (van de Peer et al. 2017), leading to evolutionary innovations and diversification (Walden et al. 2020; Ostendorf et al. 2021).

Sometimes, polyploid cells lose parts of their chromosome set, resulting in aneuploidy. For various eukaryotes, aneuploidy is mostly harmful or even lethal (Birchler and Veitia 2012; Torres et al. 2008). For example, aneuploidy is a hallmark of cancer, with about 68% of solid tumours in humans being aneuploid (Duijf et al. 2013; Passerini et al. 2016). It is well established that chromosomal instability causes aneuploidy which drives tumour formation, but there is growing evidence that aneuploidy itself might contribute to tumorigenesis (Ben-David and Amon, 2020). In humans, aneuploidy caused by the addition of one single chromosome, as extensively investigated in the chromosomal-disorder disease trisomy 21, has severe consequences and leads to characteristic phenotypical alterations. Here, the majority of genes on the multiplied chromosome 21 showed a quantitative stoichiometric 1.5 fold increase in expression (Amano et al. 2004). However, regions with altered gene expression occur all over the genome, revealing that aneuploidy affects global transcript patterns (Letourneau et al. 2014).

In contrast to aneuploids, euploid organisms deriving from a whole-genome duplication (WGD) are viable and show less phenotypical deviations. The phenotypical effects of WGDs in plants include increased cell sizes and biomass production (Wu et al. 2012; del Pozo and Ramirez-Parra 2015). Similar to aneuploidy, a WGD can result in qualitative changes in gene expression, for example by an upregulation stronger than anticipated by the increased gene dosage (Guo et al. 1996), as well as in an unaltered level of gene products, presumably caused by gene dosage compensation mechanisms (Birchler and Veitia 2012; Shi et al. 2015).

In allopolyploids with their chromosome sets originating from different taxa, a synergy between chromosome duplication and hybrid vigor or heterosis effect may occur, associated with increased growth rates, a diverging morphology and an improved ability to adapt to new environmental conditions (Comai 2005; Sattler et al. 2016). Therefore, allopolyploidization is an attractive strategy for the optimization of crop plants in agriculture (Matsuoka 2011; Behling et al. 2020), and allows them to take over new niches (Cheng et al. 2018). For example, there is molecular evidence for allopolyploidy in some mosses of the genus *Physcomitrium* which are important land pioneers (Beike et al. 2014; Medina et al. 2018). However, in autopolyploids, with chromosome sets from the same taxon, a hybrid vigor effect is lacking and hence the overall impact of a pure WGD on the genome is weaker (Spoelhof et al. 2017). It is unclear to what extent a pure WGD affects gene expression, not only quantitatively due to increased gene dosage but also qualitatively at the global level. A qualitative change in gene expression might contribute to phenotypic effects observed after artificial pure WGDs, like a smaller fruit size in autotetraploid *Hylocereus monacanthus* plants (Cohen et al. 2013) or a reduced viability in stationary phase in isogenic yeast tetraploids (Andalis et al. 2004).

In contrast to animals, land plants undergo an alteration of generations between the haploid gametophyte and the diploid sporophyte. In most cases, this alteration is heteromorphic, i.e. gametophyte and sporophyte have different morphologies. Whilst the sporophyte dominates in angiosperms, the gametophyte dominates in mosses. Thus, most mosses are haploid in the dominating stage of their life cycle (Reski 1998a), although diploid or even triploid gametophytes exist, for example in the ecologically important peat mosses (Heck et al. 2021). While the genetic regulator for the developmental switch between gametophytic and sporophytic generation has been identified in the moss Physcomitrella (Horst et al. 2016; Horst and Reski 2016), it remains unclear why these haploid plants are so successful in evolutionary terms, and not prone to excess mutations.

The discovery that Physcomitrella repairs DNA double-strand breaks (DSBs) preferably via the homologous recombination (HR) mechanism may provide an explanation for this enigma. This highly efficient HR machinery facilitates the precise and efficient integration of foreign DNA via gene targeting (GT) with success rates of up to more than 90% (Girke et al. 1998; Kamisugi et al. 2005, 2006; Schaefer and Zrÿd, 1997; Schaefer et al. 2010; Strepp et al. 1998). Subsequently, highly efficient HR was also described for the moss *Ceratodon purpureus* (Trouiller et al. 2007). In contrast, non-homologous end joining (NHEJ) is the preferred mode for the repair of DNA–DSBs in angiosperms. NHEJ relies on a protein complex comprising Ku70, Ku80, DNA-PK_CS_, XRCC4 and DNA ligase 4 (Weterings and Chen 2008), leads to a random integration pattern of a transgene in the genome, and thereby results in low GT rates (Britt and May 2003; Iiizumi et al. 2008). Hence, all attempts to establish efficient GT strategies in seed plants were not particularly successful with reported frequencies as low as 10^−4^–10^−5^ (Beetham et al. 1999; Dong et al. 2006; Okuzaki and Toriyama 2004; Zhu et al. 1999). More recently, the CRISPR/Cas9 system was successfully applied for GT in angiosperms (Steinert et al. 2016), as well as for the realization of various agronomic traits (Qi et al. 2020; Waltz 2016). However, GT rates are still low and require elaborate screening (Barone et al. 2020; Schindele et al. 2020).

It is still puzzling why HR is so efficient in some mosses. Physcomitrella is a convenient model organism to address this question since it can be easily cultivated under controlled conditions and protocols for precise genetic engineering by GT are well established (Decker et al. 2015). Its genome sequence is available, assembled and annotated, and provides evidence for at least two WGDs in its evolutionary past (Rensing et al. 2008; Lang et al. 2018), although Physcomitrella is a functional haploid (Reski 1999). Several explanations for the high GT rates have been discussed, like an altered HR mechanism compared to angiosperms encompassing slight variations in the proteins required for HR or differential expression of their encoding genes (Puchta 2002; Reski 1998b; Strotbek et al. 2013). HR-based DNA–DSB repair in Physcomitrella relies on MRE11 and RAD50 (Kamisugi et al. 2012), which are part of a protein complex binding to the ends of broken DNA strands. Targeted knock-out (KO) of the recombinase RAD51 or the SOG1-like protein SOL proved the importance of these proteins in HR and moved DNA–DSB repair to faster but non-sequence conservative repair pathways (Goffová et al. 2019; Markmann-Mulisch et al. 2007; Schaefer et al. 2010). Further, the simultaneous presence of the kinases ATM and ATR, that are also involved in the reprogramming of Physcomitrella leaf cells into stem cells after DNA damage (Gu et al. 2020), are indispensable for GT via HR (Martens et al. 2020). A number of additional proteins have been identified that are favourable but not crucial for GT, like the homology-dependent DSB end-resection protein PpCtIP (Kamisugi et al. 2016*)* and both subunits of the XPF-ERCC1 endonuclease complex involved in the removal of 3’ non-homologous termini (Guyon-Debast et al. 2019). Additionally, two RecQ helicases possess a crucial distinct function in HR and influence GT frequency, where RecQ6 is an enhancer and RecQ4 a repressor of HR (Wiedemann et al. 2018). Similarly, Polymerase Q (POLQ) acts as an inhibitor of the HR pathway (Mara et al. 2019).

Hypotheses that are more general were proposed early on: haploidy of the tissue may favour high HR (Schaefer and Zrÿd 1997), or an unusual cell-cycle arrest may be advantageous (Reski 1998b). Physcomitrella chloronema cells stay predominantly at the G2/M-boundary (Schween et al. 2003a). This cell-cycle phase may be correlated with efficient HR, as HR requires preferentially a sister chromatid as source of the homologous nucleotide sequence that is only available in the late S-phase and in the G2-phase (Heyer et al. 2010; Watanabe et al. 2009). Indeed, B1‐type CDKs and B1‐type cyclins are important regulators of HR in the angiosperm model *Arabidopsis thaliana,* linking the activity of HR to the G2-phase (Weimer et al. 2016).

A technical way to achieve GT in Physcomitrella is PEG-mediated protoplast transformation. In protoplasts, the recovery from cell-wall removal and isolation of single cells is expected to happen in the same period as the integration of the transgene via HR. This is assumed to be completed within the first 72 h after isolation before the first cell division (Xiao et al. 2012). Hence, transfected protoplasts are an interesting system to study both of these processes simultaneously. Further, analyses of protoplasts allow insights into plant defence, stress mechanisms and the regeneration of the cell wall (He et al. 2007). In Physcomitrella protoplasts, the primary cell wall was already re-established one day after isolation and after two days, they are partially reprogrammed into stem cells to re-enter the cell cycle. Finally, after three days the majority of the protoplasts have divided and developed into chloronema tissue, which is the basis for the regeneration of the whole plant (Abel et al. 1989; Xiao et al. 2012).

Here, we studied gene expression patterns in haploid and in diploid Physcomitrella plants with an artificial WGD, created by protoplast fusion (= somatic hybridization), and subsequent regeneration of diploid gametophytic plants (Schween et al. 2005a). An analysis of their cell cycle revealed no differences between haploid and autodiploid plants (Schween et al. 2005a). We focus on three sets of experiments: (i) transcriptomic changes during early phases of protoplast regeneration, (ii) transcriptomic changes that may be related to transgene integration after protoplast transfection, and (iii) consequences of a WGD for protoplast regeneration and transgene integration.

## Materials and methods

### Plant lines

In this study, a wild-type (WT) Physcomitrella (IMSC no. 40001; new species name *Physcomitrium patens* (Hedw.) Mitt., as proposed by Beike et al. 2014, Medina et al. 2019) was analysed, as well as several lines derived from it. Different haploid and diploid parental lines were derived from WT protoplasts after transformation experiments with a mutagenized cDNA library (Egener et al. 2002; Schween et al. 2005b). For the current study, plants from regenerating protoplasts were selected that had not taken up foreign DNA, as indicated by the absence of the npt II cassette (confirmed by PCR) and did not survive later treatment with antibiotics. While most regenerating plants were haploid, some were polyploid, most likely because of protoplast fusion during the PEG-treatment of the transformation procedure (Egener et al. 2002; Schween et al. 2005b). From this pool of plants, two haploid and three diploid lines were selected: Haploid A, Haploid B as well as Diploid A, Diploid B and Diploid C. Growth on Knop medium differed between the lines but with no significant influence of ploidy (Schween et al. 2005a). Flow cytometric analyses revealed that all Physcomitrella lines analysed here, whether haploid or diploid, remain predominantly at the G2 phase of the cell cycle and only few cells were in the G1 phase (Schween et al. 2005a; Supplementary Figure S1).

We used WT for the construction of a first microarray cDNA library. A second microarray experiment was carried out with WT, Haploid A, Diploid A and Diploid B. A SuperSAGE library was constructed from WT, Haploid A and Diploid A. For quantitative real-time PCR (qRT-PCR), WT, Haploid A and Diploid A were used. We analysed GT rates with Haploid A, Haploid B, Diploid A, Diploid B, and Diploid C. The characteristics of all moss lines used in this study are compiled in Table 1.

**Table 1:** Characteristics of all Physcomitrella lines used in this study

Line	IMSC number	Ploidy	Dominant cell cycle stage	Origin	Experiment
WT	40366 (WTIII) 40001 (WTIX)	haploid	G2	Wild type	1st microarray, 2nd microarray, SuperSAGE, qRT-PCR
Haploid A	40369	haploid	G2	Regenerating transformed protoplasts	2nd microarray, SuperSAGE, qRT-PCR, GT rate
Haploid B	40368	haploid	G2	Regenerating transformed protoplasts	GT rate
Diploid A	40371	diploid	G2	Regenerating transformed protoplasts	2nd microarray, SuperSAGE, qRT-PCR, GT rate
Diploid B	40370	diploid	G2	Regenerating transformed protoplasts	2nd microarray, GT rate
Diploid C	40873	diploid	G2	Regenerating transformed protoplasts	GT rate

The cell cycle stage was determined with flow cytometry in Schween et al. (2005a)

### Cell culture conditions

For transformation, the moss lines were cultivated in liquid or on solid modified Knop medium according to Reski and Abel (1985). The material for microarrays, SuperSAGE and qRT-PCR was grown in liquid Knop medium supplemented with microelements (Egener et al. 2002). Cultivation and protoplast isolation were performed as described in Frank et al. (2005), which is based on highly standardized and efficient procedures developed by us (Hohe et al. 2004).

### Transformation

The GT construct pRKO25.2 (Hohe et al. 2004) contains a 1920 bp cDNA fragment of the cold-responsive gene Pp3c21_180V3, encoding sphingolipid fatty acid desaturase (PpSFD) (Beike et al. 2015; Resemann et al. 2021). This construct contains the coding sequence for neomycin phosphotransferase (npt II) driven by a Nopaline synthase (NOS) promoter and terminator. Transformation, selection and regeneration were performed as described in Frank et al. (2005). Selection was done twice for 2 weeks on medium containing 25 µg/ml G418 (Promega, Mannheim, Germany) starting 2 weeks after transformation with a 2-week release period in between. Preparation of material for subsequent RNA isolation and microarray or SuperSAGE analyses was performed with 300,000 protoplasts at different time-points after isolation and transfection with 20 µg of pRKO25.2 construct per time-point.

### PCR analysis

All PCR primers are listed in Supplementary Table T1, a schematic overview of primer locations is given in Supplementary Figure S2. PCR-based analysis of the transgenics was performed according to Schween et al. (2002). The primer combination npt2cdc1-L and npt2cdc1-R was used to detect the npt II cassette. The primers JMLKO25L and JMLKO25R were used to amplify a specific endogenous fragment of 295 bp in WT plants. Homologous integration of the construct into the endogenous genomic locus was monitored using primers JMLKO25-L3 and JMLK2-R5, which were derived from the border of the npt II cassette and the border of the genomic locus at the 5’ end and with primers JMLK2-F3 and JMLKO25-L4, which were derived from the border of the npt II cassette and the border of the genomic locus at the 3’ end, respectively. Two to four independent samples from each plant were tested with all primers to ensure correct identification of KO plants. The significance of ploidy on the transformation results was evaluated with Fisher’s exact test.

### RNA isolation and cDNA synthesis

Total RNA for microarray and SuperSAGE studies was isolated from protonema and protoplasts using the RNeasy Plant Mini Kit (Qiagen, Hilden, Germany), applying on-column DNA digestion with DNaseI in accordance with the manufacturer’s protocol. Isolation of total RNA for qRT-PCR was performed in the same manner with protonema as starting material. The DNA digestion was performed as a separate step with DNaseI (ThermoScientific, Darmstadt, Germany) after purification of RNA. cDNA was synthesised using the TaqMan Reverse Transcription Reagents Kit (ThermoScientific) according to the manufacturer’s protocol with oligo(dT) primers. For each of the three technical replicates, cDNA corresponding to 50 ng of total RNA per transcript was used for quantification. A non-transcribed (-RT) control was included to confirm successful DNA digestion. Primers (Supplementary Table T1) were designed with the help of the Roche Life Science Universal Probe Library Assay Design Center (https://lifescience.roche.com). Prior to further analyses, a melting curve analysis was performed for each primer pair. qRT-PCR of protonema samples was conducted using the SensiFastTM SYBR No-ROX Kit (Bioline, Luckenwalde, Germany) in a LightCycler 480 (Roche, Mannheim, Germany). For normalization of variations in cDNA content the reference genes encoding EF1α (Pp3c2_10310V3.1) and TBP (Pp3c12_4720V3.1) were used (Richardt et al. 2010). The relative transcript abundance was calculated in relation to the reference genes with a modified ΔΔC_t_ approach as described in Hellemans et al. (2007).

### Microarray experiments

The microarray experiments were performed with a 90 K whole genome microarray (Combimatrix Corp., Mukilteo, WA, USA) as described previously (Beike et al. 2015; Kamisugi et al. 2016; Wolf et al. 2010). For each time-point per biological replicate, 1.5 µg of RNA were transcribed into cDNA and amplified to aRNA. Subsequently, 5 µg of aRNA were labelled with Cyanine-5 (RNA ampULSe: amplification and labelling kit; Kreatech, Amsterdam, the Netherlands). The resulting labelled aRNA was fragmented (Fragmentation Reagents; Ambion, Austin TX, USA) and hybridised overnight to the microarray following the manufacturer’s instructions. Visualization was performed with a laser scanner (Genepix 4200A; Molecular Devices, Ismaning, Germany) and images were analysed with the Microarray Imager 5.9.3 Software (Combimatrix Corp.). All time-points were analysed in three biological replicates. The microarray slides were stripped with a stripping kit (Combimatrix Corp.) and reused up to four times. The experimental procedure was the same as described previously (Beike et al. 2015; Kamisugi et al. 2016; Wolf et al. 2010).

### Microarray data analysis

Microarray expression values were investigated with the Expressionist Analyst Pro software (v5.0.23, Genedata, Basel, Switzerland). The probe sets were median condensed, and linear array-to-array normalization was applied using median normalization to a reference value of 10,000. Differentially expressed genes were detected using the Bayesian regularised unpaired CyberT test (Baldi and Long 2001) with Benjamini–Hochberg false discovery rate correction and a minimum |log_2_ fold change|> 1 (Richardt et al. 2010). A false discovery rate of *q* < 0.05 was taken as cut-off for the first microarray time series experiment. For the second microarray time series experiment *p* < 0.001 was chosen for the comparison of gene expression between the ploidy levels and for the comparison of gene expression between different time-points in regenerating protoplasts. K-means clustering with *k* = 2 identified upregulated and downregulated genes. An overview of the plant lines and sample sources used for the different comparisons to compute DEGs is compiled in Supplementary Table T2.

### SuperSAGE library construction

SuperSAGE libraries were constructed by GenXPro (Frankfurt am Main, Germany) following a protocol based on Matsumura et al. (2010) as described by El Kelish et al. (2014) with the implementation of GenXPro-specific technology and improved procedures for quality control as well as specific bias proved adapters for elimination of PCR artefacts (True-Quant methodology). In total, 17 SuperSAGE libraries (including replicates) were constructed from 11 biological samples. The biological samples encompass: The transcriptome of Haploid A and Diploid A after protoplast isolation (0 h) and 4 h and 24 h after transfection; haploid as well as diploid protonema mRNA in duplicates; transcript data of WT protoplast from 0 h, 4 h and 24 h with triplicates for 4 h and 24 h. A detailed overview of the libraries is provided in Supplementary Table T3.

### SuperSAGE data analysis

The quality of the processed libraries was checked with FastQC (v0.11.4, Andrews, 2010) and reads were mapped with HISAT2 (v2.0.3, Kim et al. 2015) to the V3 assembly of the *P. patens* genome (Lang et al. 2018) in the Galaxy platform (Freiburg Galaxy instance, http://galaxy.uni-freiburg.de, Afgan et al. 2016). Mapping parameters allowed for no mismatches and only known splice sites were considered. A count table was constructed from the mapped reads using the featureCounts (v1.4.6.p5, Liao et al. 2014) tool from the Galaxy platform by counting all the reads mapped to exons or untranslated regions of each gene. Multiple alignments of reads were allowed, while reads with overlaps on the meta-feature (gene) level were disregarded for the construction of the count table. For specific parameters, see Supplementary Table T4 and Supplementary Table T5. Statistical analysis for differential gene expression was performed by pairwise comparison of library count tables using GFOLD (v1.1.4, Feng et al. 2012) and by two two-factor analyses with the DESeq2 package in Galaxy with default parameters (Galaxy Version 2.11.40.6, Love et al. 2014). In the two-factor analyses, ploidy-dependent gene expression was determined in the presence of tissue as secondary factor. All libraries originating from protonema and different protoplast material were used as input for the first two-factor analysis and the libraries of mock transformed WT protoplasts at 4 h and 24 h were considered as replicates to the libraries of transformed WT protoplasts at the corresponding time-points. Only libraries derived from protoplasts of the lines WT and Diploid A were considered for the second two-factor analysis. In GFOLD analysis, genes with a GFOLD(0.01) value (representing the log2 fold change of gene expression adapted for adjusted *p* value, Feng et al. 2012) of <  − 1 or > 1 were considered to be differentially expressed whereas in DESeq2 analysis genes with a |log2 fold change|> 1 and an adjusted *p* value < 0.1 were considered as differentially expressed. Further data exploration was performed using functions from SAMtools (v1.3.1, Li et al. 2009).

### Computational analysis of DEGs

Annotation of DEGs was obtained using Phytozome (v12.1.5, Goodstein et al. 2012) and the PpGML DB (Fernandez‐Pozo et al. 2020). For the computation of the overlap between DEGs identified in the microarray and SuperSAGE data, and to generate a combined set of DEGs comprising all DEGs from both technologies, gene IDs of DEGs identified in the second microarray experiment were converted to Physcomitrella V3.3 IDs (Lang et al. 2018). If one ID mapped to several genes of the V3.3 annotation all of them were considered as DEGs. In case the IDs of multiple DEGs mapped to the same V3.3 ID the mean of the log2 fold change values was taken. Similarly, in the comparison between the DEGs identified in our study and DEGs found by Xiao et al. (2012), Physcomitrella V1.6 IDs were translated into Physcomitrella V3.3 IDs. In both cases, genes with no correspondence in the V3.3 annotation were neglected. This amounted to a maximum of 50 out of 2245 DEGs that were not analysed further. Only genes that are contained in the main V3 genome according to the annotation file downloaded from PpGML DB were included in the lists of DEGs presented here. Expression data of specific genes in different developmental stages of Physcomitrella were obtained from the PEATmoss website (Fernandez‐Pozo et al. 2020). Genes that are relevant for DNA–DSB repair or for DNA repair in general were identified using biological process (PB) GO terms from the current V3.3 annotation obtained from PpGML DB and an in-house list with repair-relevant genes. The Principle Component Analysis (PCA) of SuperSAGE libraries, the gene ontology (GO) enrichment analysis and the visualization of the word cloud with enriched GO terms were carried out in R (v3.6.3, R core team, 2020). For the PCA the R package DESeq2 (v1.24.0; Love et al. 2014) was used, the word cloud with enriched GO terms was created with the R package tagcloud (v0.6, Weiner, 2015) and the word size scales with the negative log2 of the adjusted p value. The GO enrichment analyses were performed with the R package clusterProfiler (v3.12.0, Yu et al. 2012) using a *p* value cut-off of 0.005 and a *q* value cut-off of 0.2. The minimal size of genes annotated by ontology term for testing (minGSize) was set to 1 and the maximal size of genes annotated for testing (maxGSize) was set to 1000. As universe for microarray data, all genes on the microarray were taken, whereas the universe for data from SuperSAGE contained all genes of the main Physcomitrella V3 genome. Redundant GO terms were removed afterwards using the *simplify* method from clusterProfiler with default parameters. Generation of several figures and processing of tables with DEGs was performed in Python3 (v3.8.5, Van Rossum and Drake 2009) using the packages Matplotlib (v3.2.1, Hunter, 2007), NumPy (v1.18.4, Harris et al. 2020), pandas (v1.0.3, McKinney, 2010; Reback et al. 2020), rpy2 (v3.3.3, Gautier 2010), seaborn (v0.10.1, Waskom et al. 2020), and pyvenn (https://github.com/tctianchi/pyvenn).

## Results

### Regenerating protoplasts exhibit a time-dependent gene expression pattern

We generated a transcriptomic time series using microarrays to investigate how gene expression is adjusted during regeneration of transfected Physcomitrella protoplasts, and to identify those time-points during protoplast transformation with the strongest alterations in gene expression. We assume that transformation of the genome (= integration of the heterologous DNA) is completed before the first cell division of the protoplast, that happens under our conditions within the first 72 h. The 90 K whole genome microarray used here represents all Physcomitrella gene models of the genome assembly V1.2 (Rensing et al. 2008). Data were generated for WT samples at six time-points: freshly isolated (0 h) protoplasts and protoplasts 1 h, 4 h, 6 h, 24 h and 72 h after transfection.

**Fig. 1:**
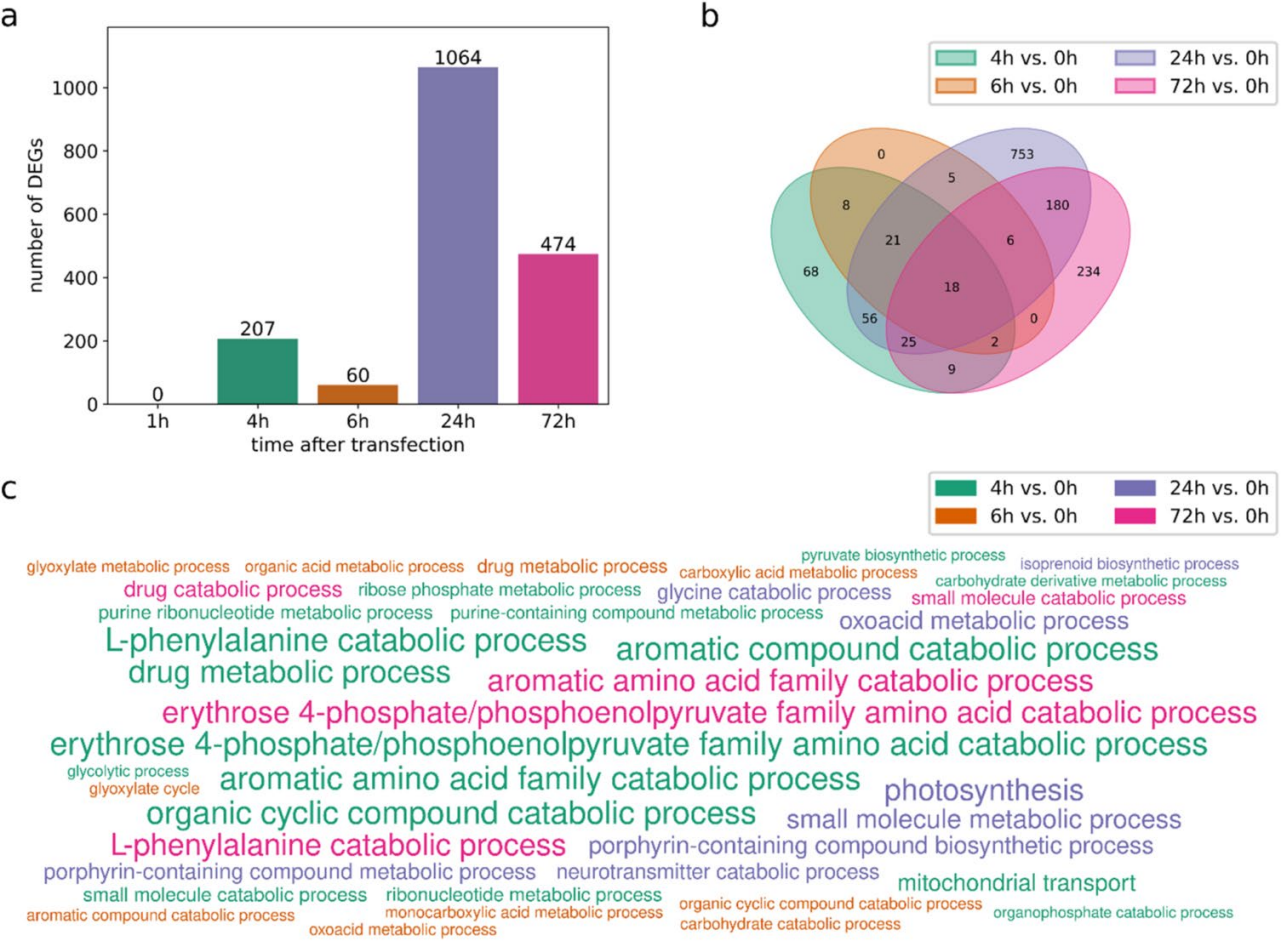
Time series of DEGs in Physcomitrella WT protoplasts 1 h, 4 h, 6 h, 24 h and 72 h after transfection compared to freshly isolated (0 h) protoplasts based on microarray data. DEGs are filtered for *q* < 0.05. (**a**) Number of DEGs at each time-point. Two maxima of DEGs are apparent after 4 h and 24 h, respectively. (**b**) Overlap of DEGs from each time-point. (**c**) Significantly enriched biological process GO terms (*p* < 0.005, *q* < 0.2). The word size scales with the negative log2 of the adjusted *p* value

A pairwise comparison to 0 h revealed two maxima of differentially expressed genes (DEGs) at 4 h (207 DEGs) and 24 h (1064 DEGs, Fig. 1a), whereas gene expression was unaltered at 1 h. Most DEGs at 6 h were also DEGs at 4 h (Fig. 1b). These include genes encoding glyceraldehyde-3-phosphate dehydrogenase (Pp3c21_9380), a key player in glycolysis, malate synthase (Pp3c20_22510) and isocitrate lyase (Pp3c7_2470), which are enzymes of the glyoxylate cycle (Supplementary Excel sheet1). At 24 h, 933 additional DEGs that were not identified at earlier time-points were detectable. 10 of the DEGs detected at 4 h and 6 h were not significantly differentially expressed at 24 h anymore. Nearly half of the DEGs at 72 h were specific for this time-point. However, 9 genes that were exclusively differentially expressed at 4 h but not 6 h and 24 h were now again among the DEGs, for example the genes encoding 4-coumarate-CoA ligase (Pp3c19_13170) and phenylalanine ammonia lyase (Pp3c2_30610). 18 out of the total 1385 DEGs were constantly differentially expressed at each time-point between 4 and 72 h, three of them being associated with the plant hormone gibberellin that regulates developmental processes, also in Physcomitrella (Vandenbussche et al. 2007). These are Pp3c5_4920 and Pp3c4_2230, encoding 2-oxoglutarate and Fe(II)-dependent oxygenases involved in gibberellin biosynthesis, as well as Pp3c1_15680 that encodes a homologue to the Arabidopsis GRAS family protein RGL1. We also detected DEGs related to auxin, ABA and jasmonic acids, at up to three time-points. Another gene with a constant differential regulation was Pp3c19_6540, which encodes a catalase that acts against oxidative stress by degradation of H_2_O_2_. Several other stress-related genes were differentially expressed at one specific or multiple time-points during protoplast regeneration.

To gain a deeper insight into protoplast regeneration, we performed a gene ontology (GO) enrichment analysis for those time-points where DEGs were identified (Fig. 1c). We considered terms with *p* < 0.005 and *q* < 0.2 as significantly enriched and reduced the number of resulting GO terms by selecting a representative term amongst similar terms. A strong enrichment of genes associated with erythrose 4-phosphate/phosphoenolpyruvate family amino acid catabolic process, l-phenylalanine catabolic processes and aromatic amino acid family catabolic processes was observed at 4 h and at 72 h (Supplementary Excel sheet 2). Only at 4 h, genes with the GO term glycolytic process were enriched, while the GO terms drug metabolic process, organic cyclic compound catabolic process and aromatic compound catabolic process that were strongly enriched at 4 h were also enriched at 6 h, however to a lower extent. Other enriched GO terms at 6 h were carbohydrate catabolic processes and glyoxylate cycle. At 24 h, mostly the expression of genes with the GO-term photosynthesis altered. Other strongly enriched GO terms in the DEGs at 24 h were porphyrin-containing compound biosynthetic process, oxoacid metabolic process and small molecule metabolic process. Further, we found an enrichment of ammonia-lyase activity and ammonia-ligase activity at 4 h and 72 h, while we observed an enrichment of aminomethyltransferase activity at 24 h.

### A high number of DEGs in haploid and diploid protoplasts at 24 h

**Fig. 2:**
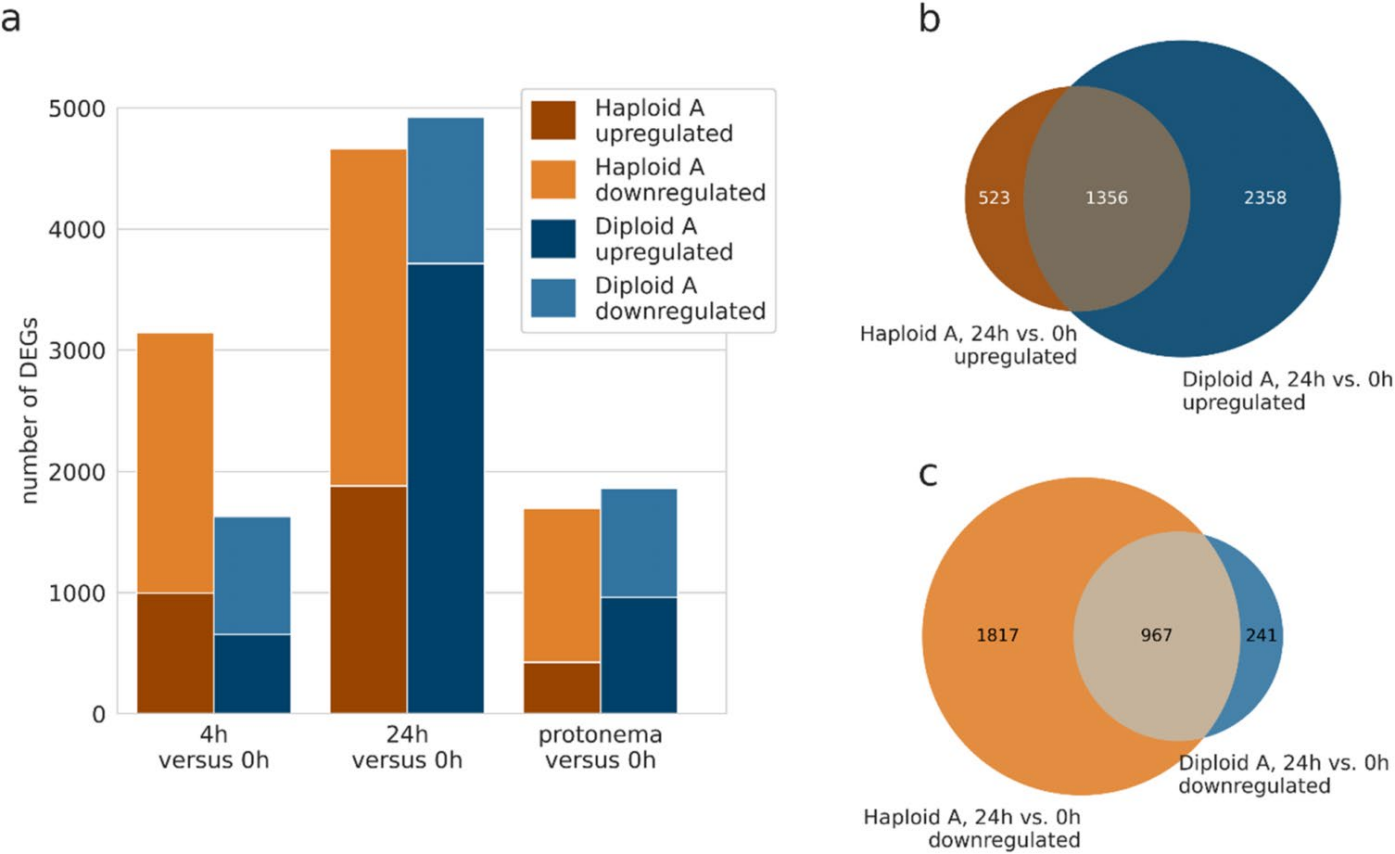
Number of genes being upregulated or downregulated in protoplasts 4 h and 24 h after transfection as well as protonema (PN) compared to freshly isolated protoplasts (0 h). (**a**) The number of DEGs identified in one haploid line (Haploid A, brown) and in one diploid line (Diploid A, blue). DEGs are combined from identification in the microarray data (filtered for a |log2 fold change|> 1 and *p* < 0.001) and the SuperSAGE data (filtered for a GFOLD(0.01) value of < −1 or > 1). (**b**) Overlap between the upregulated genes at 24 h in the haploid line with the upregulated genes at 24 h in the diploid line. (**c**) Overlap between the downregulated genes at 24 h in the haploid line with the downregulated genes at 24 h in the diploid line

To investigate if haploid and diploid protoplasts behave differently during regeneration, we generated transcriptomic data of two haploid and two diploid lines. We analysed protonema, untransformed protoplasts (0 h), and protoplasts at 4 h and 24 h (Fig. 2, Supplementary Table T6). For a more detailed follow-up analysis, we additionally applied the SuperSAGE sequencing technology. Compared to microarrays, SuperSAGE has the advantage that sampling is based on sequencing rather than hybridization of RNA and as a consequence, sequences do not need to be known a priori. Furthermore, sequenced reads can be directly mapped to the genome and gene expression is quantified by direct counts of transcript abundance, thus eliminating background noise that exists in microarrays. This leads to increased sensitivity and improved gene expression quantification. Altogether, we generated 17 SuperSAGE libraries (Supplementary Table T3). Four libraries (2 WT samples at 4 h and 24 h respectively) were derived from mock transformants subjected to the whole transfection procedure but using water instead of the GT construct. Pairwise comparison between SuperSAGE libraries was performed with GFOLD, an algorithm especially developed for approaches when only few replicates are available.

Time-dependent DEGs were computed with samples taken from protoplasts at 4 h or 24 h versus protoplasts at 0 h, separately for haploids and diploids (Supplementary Table T6). Neither in haploids nor in diploids had we found an extensive overlap between the DEGs from microarrays and SuperSAGE showing the advantage of combining both methods (Supplementary Figure S3). We observed for the haploid WT 1148 DEGs at 4 h and 4000 DEGs at 24 h (SuperSAGE) and for the line Haploid A 3142 DEGs at 4 h and 4663 DEGs at 24 h (combination of DEGs from microarray and SuperSAGE). This makes a combined set of DEGs from both haploid lines with 3823 DEGs at 4 h and 6698 DEGs at 24 h (467 DEGs at 4 h and 1965 DEGs at 24 h, respectively, were found in both haploid lines, Supplementary Figure S3). Considering the total number of Physcomitrella protein-encoding genes (32,458 in Physcomitrella V3.3, Fernandez-Pozo et al. 2020; Lang et al. 2018) 3.54% and 12.32% of them were differentially expressed in WT at 4 h and 24 h, respectively, while it was 9.68% at 4 h and 14.37% at 24 h in Haploid A. For Diploid A, we received from microarray and SuperSAGE a set of in total 1628 DEGs at 4 h and 4922 DEGs at 24 h, being 5.02% and 15.16%, respectively, of the Physcomitrella genes. According to the GO analysis in our initial microarray time series (Fig. 1c) we identified in the subsequent microarray data of Haploid A an enrichment of photosynthesis-related genes at 24 h and additionally at 4 h, but they were not enriched in the data of Diploid A. However, in the SuperSAGE data of both haploids and diploids photosynthesis-related genes were enriched at 24 h as well as at 4 h (Supplementary Excel sheet 2). In the comparison of 24 h versus 0 h, the percentage of downregulated DEGs was noticeably lower in microarrays and SuperSAGE of the diploid line than in the haploid lines (Fig. 2, Supplementary Table T6). For the haploid lines the percentage of downregulated DEGs at 24 h was in Haploid A 39.95% and 73.66% in microarray and SuperSAGE data, respectively, and 70.83% in the SuperSAGE data of WT whereas for Diploid A 16.84% of the DEGs at 24 h were downregulated in the microarray data and 26.44% in the SuperSAGE data. In each of the analysed data sets more than half of the upregulated DEGs at 24 h in the haploid lines were also upregulated in the diploid line (e.g., 72.17% for Haploid A; Fig. 2b). Similarly, most of the downregulated DEGs at 24 h in the diploid line were also downregulated in the haploid lines (e.g., 80.05% compared to Haploid A; Fig. 2c).

### Combination of microarray and SuperSAGE sequencing yields a high amount of new DEGs

Xiao et al. (2012) investigated regenerating Physcomitrella protoplasts and their reprogramming into stem cells at four time-points: 0 h, 24 h, 48 h and 72 h. We looked for similarities and differences in the DEGs identified from the 24 h versus 0 h comparison in that study and the DEGs computed from protoplast samples from 24 h after transfection versus 0 h using the combined set of microarray and SuperSAGE data from two haploid lines (WT, Haploid A) in our current study (Supplementary Table T6). Of the 1195 DEGs at 24 h versus 0 h from Xiao et al. (2012) that were translated into Physcomitrella V3.3 IDs (5 of the 1095 DEGs did not map to any V3.3 ID; 115 mapped to multiple IDs), 801 (69.71%) were also identified in at least one of our datasets of the haploids (Supplementary Figure S3), whereas we identified 5898 additional DEGs. Among the 394 genes that were only found to be differentially expressed by Xiao et al. (2012), some with high log2 fold changes in expression of > 5 or <  − 5 are for example a homologue to an Arabidopsis threonine aldolase (Pp3c4_31180) and a cytochrome P450 (Pp3c11_6580). Genes with high expressional changes at 24 h that were only found in our data are amongst others a gene encoding a desiccation-related protein of the LEA family (Pp3c17_8560), and an ap2 erf domain-containing transcription factor (Pp3c9_4590). In Xiao et al. (2012), 65.02% of the DEGs were downregulated at 24 h versus 0 h. According to our SuperSAGE data of both haploid lines these are 70.83% and 73.66%, respectively, while according to our microarray data of Haploid A only 39.95% DEGs were downregulated (Supplementary Table T6).

### Key players of HR and NHEJ are differentially expressed in haploid and diploid lines during protoplast regeneration

In total, we identified 255 genes that are relevant for DNA repair among the combined set of DEGs at 4 h or 24 h (Supplementary Excel sheet 1). Most of those that act in HR or NHEJ, the two main DNA–DSB repair pathways, were upregulated. In WT and Haploid A we identified 29 HR-relevant DEGs and 30 in Diploid A. 18 of them occurred in haploids and diploids (Supplementary Excel sheet 1). For some, differential expression occurred in haploids and diploids at different time-points during protoplast regeneration. For example, the gene encoding the HR-relevant protein Rad50 (Pp3c10_3760; Kamisugi et al. 2012) was upregulated specifically, whereas upregulation in haploids occurred exclusively at 4 h, it occurred in diploids only at 24 h. In contrast, we observed at 24 h in both ploidies a time-dependent upregulation of REV1 (Pp3c22_4740), a HR-promoting protein (Sharma et al. 2012). Six DEGs encode proteins with a role in NHEJ (Supplementary Excel sheet 1) with XRCC4 (Pp3c1_38430), Ku70 (Pp3c18_7140) and Ku80 (Pp3c22_11100) as the key proteins of NHEJ (Weterings and Chen 2008). Their expression was upregulated in both ploidies. The upregulation of XRCC4 was consistent over time at 4 h and 24 h, whereas the upregulation of Ku70 and Ku80 started only at 24 h. The other NHEJ-related genes were all exclusively upregulated at 24 h. These are PRKDC (Pp3c9_15240), POLL (Pp3c15_19010) and ATM (Pp3c2_23700). PRKDC encodes a protein kinase that is recruited to the ends of DNA by the Ku70/Ku80 complex (Davis and Chen 2013). POLL encodes Polymerase λ that plays a role in gap-filling (Lee et al. 2004). ATM encodes a protein kinase that is activated by DNA–DSBs, triggering cell-cycle checkpoint signalling and DNA repair (Maréchal and Zou 2013). ATM plays a role in NHEJ and in HR (Bakr et al. 2015; Weterings and Chen 2008; Zha et al. 2011) and was the only NHEJ-related DEG that was differentially expressed in diploids but not haploids. Further, we found genes encoding proteins of other DNA-repair pathways like nucleotide excision repair and base excision repair (Supplementary Excel sheet 1). Interestingly, the second XRCC4-like gene in the Physcomitrella genome (PP3c14_21160) was not detected as DEG in our studies.

### Gene expression in diploid and haploid protoplasts differs at various time-points

Next, we determined DEGs between haploids and diploids separately for 0 h, 4 h, 24 h, and in protonema. In the microarray data only few DEGs were identified (Table 2, column DEGs in microarray; Supplementary Table T7) and the differences in expression levels were small (Supplementary Excel sheet 1).

**Table 2:** Number of DEGs between diploid and haploid cells at different time-points after transfection

Sample	DEGs in microarray	DEGs in SuperSAGE	Overlap of DEGs in microarray and SuperSAGE
0 h protoplasts	36	426	18
4 h protoplasts	88	2328	21
24 h protoplasts	43	3010	21
Protonema	67	0	0

Microarray and SuperSAGE analysis were performed on cells from Diploid A compared to cells from Haploid A. DEGs from the microarray experiment were determined with the Expressionist Analyst Pro software and were filtered for |log2 fold change|> 1 and *p* < 0.001. The SuperSAGE data analysis was performed with GFOLD and DEGs were filtered for a GFOLD(0.01) value of <  − 1 or > 1

**Fig. 3:**
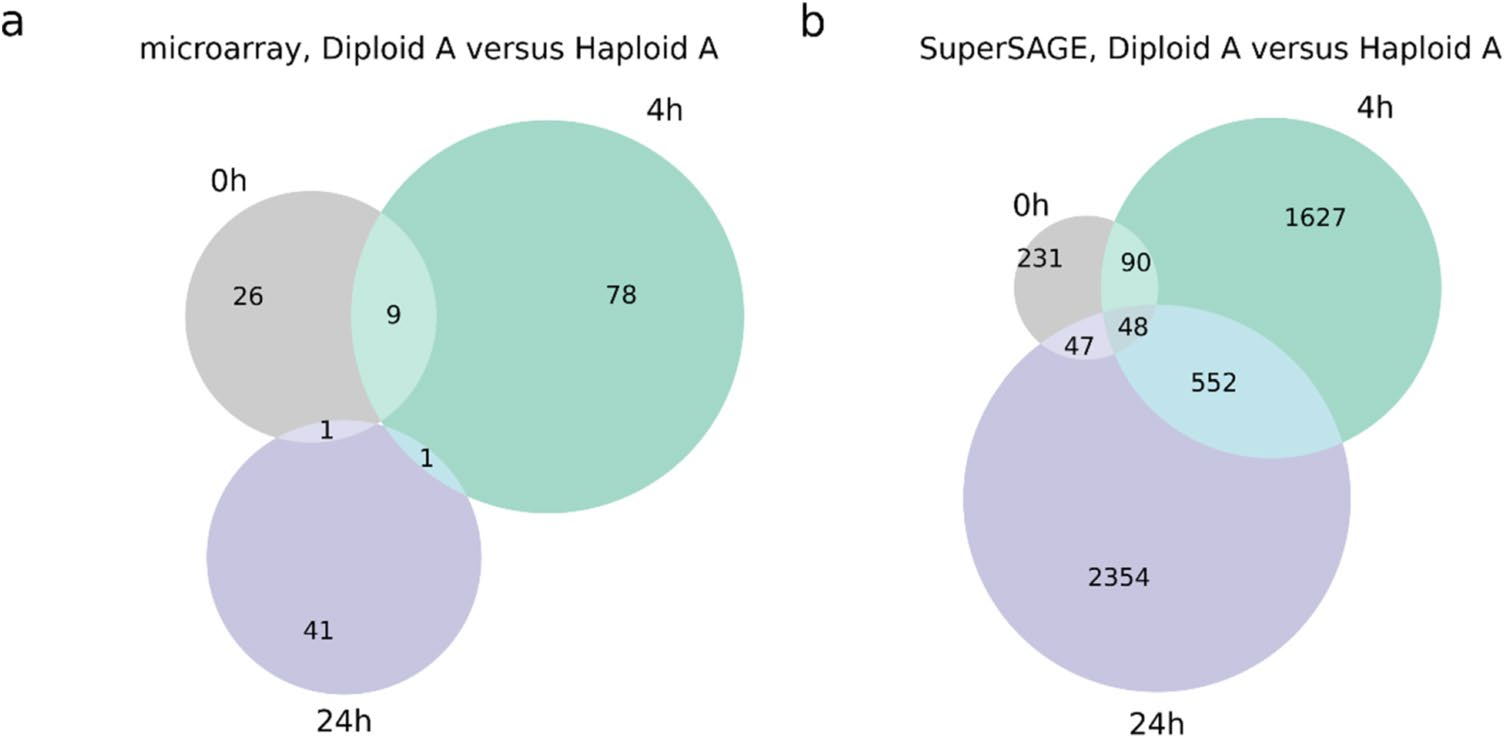
Overlap of DEGs identified from pairwise comparison between one haploid (Haploid A) and one diploid (Diploid A) line at different protoplast stages. DEGs were determined by microarray analysis (**a**) and SuperSAGE libraries (**b**) from protoplast samples (grey: freshly isolated protoplasts (0 h), green: protoplasts 4 h after transfection, purple: protoplasts 24 h after transfection. DEGs from the microarray experiment were determined with the Expressionist Analyst Pro software and were filtered for |log2 fold change|> 1 and *p* < 0.001. The SuperSAGE data analysis was performed with GFOLD and DEGs were filtered for a GFOLD(0.01) value of <  − 1 or > 1

Further, we performed pairwise comparisons between haploid and diploid SuperSAGE libraries (Table 1). The pairwise SuperSAGE data analysis of protonema samples from Diploid A versus Haploid A yielded no DEGs while it was 67 in the microarray analysis of the same lines. In contrast, we found more pronounced ploidy-dependent transcriptomic differences in the SuperSAGE data of protoplasts than in the microarray data (Table 2, Supplementary Table T7). There was a moderate overlap between DEGs in microarray and SuperSAGE data (Table 2, Supplementary Figure S4). Most of the DEGs were specific to a certain time-point, while only few of them were common among all protoplast stages (48 in SuperSAGE, 0 in microarray; Fig. 3). Examples are some membrane and surface proteins (e.g., Pp3c3_22320 and Pp3c6_11380) as well as an oxidoreductase (Pp3c4_15600). The same trend that the majority of DEGs only appeared at one specific time-point occurred also in pairwise comparisons using additionally Diploid B and WT (Supplementary Figure S4, Supplementary Excel sheet 1). There were noticeable differences in the number and identity of DEGs between analyses with different combinations of plant lines (Supplementary Figure S4). An additional principle component analysis (PCA) of the 17 SuperSAGE libraries revealed that the plant material (protonema or protoplast) and the protoplast regeneration states (0 h, 4 h, 24 h) had the strongest contribution to the variance in gene counts between the libraries, whereas the contribution of ploidy was inferior (Supplementary Figure S5). Besides, at 4 h and 24 h there was a considerable variance in the gene counts between the two haploid lines.

### Ploidy affects expression of 3.7% of the Physcomitrella genes

To gain more insight into ploidy-specific gene expression, we performed two analyses with a two-factor design. This was set up to test for the changes in gene expression caused by ploidy taking the impact of tissue and time into consideration. This design allowed the inclusion of all SuperSAGE datasets in a ploidy-dependent differential gene expression analysis, resulting in a more robust test. The tool for these investigations was DeSeq2, which is suitable for multiple-factor analysis with few replicates (Love et al. 2014). For the first analysis, all 17 SuperSAGE libraries from WT, Haploid A, and Diploid A were used (Supplementary Table T3). In a second analysis, we used the same two-factor design, but exclusively compared datasets derived from protoplasts of Diploid A with protoplasts of the haploid WT control, omitting the protonema samples to ensure a more homogenous data collection. In the first analysis, we identified 159 ploidy-dependent DEGs (89 upregulated and 70 downregulated in diploids). The second analysis yielded 1170 DEGs (635 upregulated and 535 downregulated in diploids). Combining the genes with ploidy-dependent expression from both analyses resulted in a total set of 1202 DEGs (127 DEGs were identified in both analyses), comprising 3.7% of the Physcomitrella protein-encoding genes (32,458 in V3.3; Fernandez‐Pozo et al. 2020; Lang et al. 2018). The rate of alteration in expression was mostly moderate; differences of more than eightfold (|log2 fold change|> 3) were detected only rarely (5 upregulated, 1 downregulated) and exclusively in the second two-factor analysis (Supplementary Table T8). We found the highest upregulation for a gene encoding a glycosyl-hydrolase-family 88 protein (Pp3c23_940), which cleaves saccharide bonds (Davies and Henrissat 1995). The gene with the highest downregulation encodes a small subunit ribosomal protein S27a (Pp3c4_19000) that can play a role in disease resistance and cell death (Xu et al. 2019). Other genes with changes of more than eightfold encode a chalcone-flavonone isomerase 3 related protein (Pp3c4_25770), a 3-hydroxyisobutyryl-Co A hydrolase (Pp3c22_10130), and two unannotated genes (Pp3c4_17790 and Pp3c2_21940). Two of the most prominently downregulated genes from the first two-factor analysis (Supplementary Table T9) encode proteins related to cell wall organization: A BR-signalling kinase (Pp3c20_8180; Rao and Dixon, 2017) and an expansin (Pp3c3_16280; Marowa et al. 2016; Schipper et al. 2002).

### Ploidy-dependent expression of genes involved in plant morphogenesis, cell cycle regulation, DNA–DSB repair and DNA accessibility

Altered phenotypes after a WGD in several species (Andalis et al. 2004; Cohen et al. 2013) motivated us to look for differential regulation of genes that might contribute to phenotypic alterations between haploid and diploid Physcomitrella lines. In the DEGs from the two-factor analyses we searched for moderate to strong differences with a |log2 fold change|> 1.5 and a biological process GO term developmental process (GO:0,032,502), growth (GO:0,040,007) or any of their child terms. The search yielded 9 DEGs (Supplementary Table T10), including a MAPKKK (Pp3c1_10860), a protochlorophyllide oxidoreductase (Pp3c22_2330) and a pectate lyase (Pp3c13_1640).

Subsequently, we performed a targeted search in the pairwise time-point-specific comparisons between the ploidies and the results from the two-factor analyses for differential expression of genes with a role in DNA–DSB repair, cell cycle regulation and DNA accessibility. In the combined set of DEGs, we observed that most HR- and NHEJ-related genes were upregulated in diploids compared to haploids (Supplementary Excel sheet 1). From the 21 HR-relevant DEGs only 3 were downregulated in diploids. These encode the ATPase YcaJ (Pp3c23_21270), DNA polymerase I (Pp3c14_14550) and poly(ADP-ribose) polymerase (Pp3c8_13220). Only 3 NHEJ-related ploidy-dependent DEGs were identified; all of them upregulated. They encode ATM (Pp3c2_23700), the DNA-dependent protein kinase catalytic subunit (Pp3c9_15240) and XRCC4 (Pp3c1_38430). The latter two are known players of NHEJ (Brouwer et al. 2016; Chang et al. 2017; Graham et al. 2016). Additionally, the gene encoding Polymerase Q (POLQ) was upregulated (Pp3c5_12930). It acts in alternative end-joining and is an inhibitor of HR in Physcomitrella (Mara et al. 2019).

From these, XRCC4 was the only one that was also a DEG in one of the two-factor analyses, representing the gene with the strongest transcriptomic difference between haploids and diploids that is directly involved in DNA repair. Hence, we chose XRCC4 for validation with qRT-PCR. Furthermore, three genes with a function in cell-cycle regulation or DNA accessibility were selected from the upregulated DEGs of the two-factor analyses (Table 3): CENPE (Pp3c22_20430), cyclin D2 (Pp3c9_8300) and H3K4-Methyltransferase (Pp3c4_16880).

**Table 3:** Overview of ploidy-dependent expressed genes with reported functions in DNA–DSB repair, cell cycle regulation and DNA accessibility identified by two-factor analyses

Gene name/Gene ID	Biological function	DESeq2 analysis	Log2 fold change
CENPE Pp3c22_20430	Centromere-associated protein E-homolog, kinesin domain, mostly chromatin silencing, in mammals connected to G2 phase of the cell cycle (Abrieu et al. 2000)	First analysis Second analysis	1.04 1.18
Cyclin D2 Pp3c9_8300	Cell-cycle regulation: G1/S phase transition	Second analysis	1.41
H3K4 MET Pp3c4_16880	Histone lysine (H3K4) methyl-transferase, mostly chromatin activating	Second analysis	1.06
XRCC4 Pp3c1_38430	X-ray repair cross-complementing protein 4-homolog, DNA–DSB repair via NHEJ pathway	Second analysis	1.76

Expression fold changes are given for diploid cells in comparison to haploid cells

### qRT-PCR validates upregulation of XRCC4 in diploid protonema cells

**Fig. 4:**
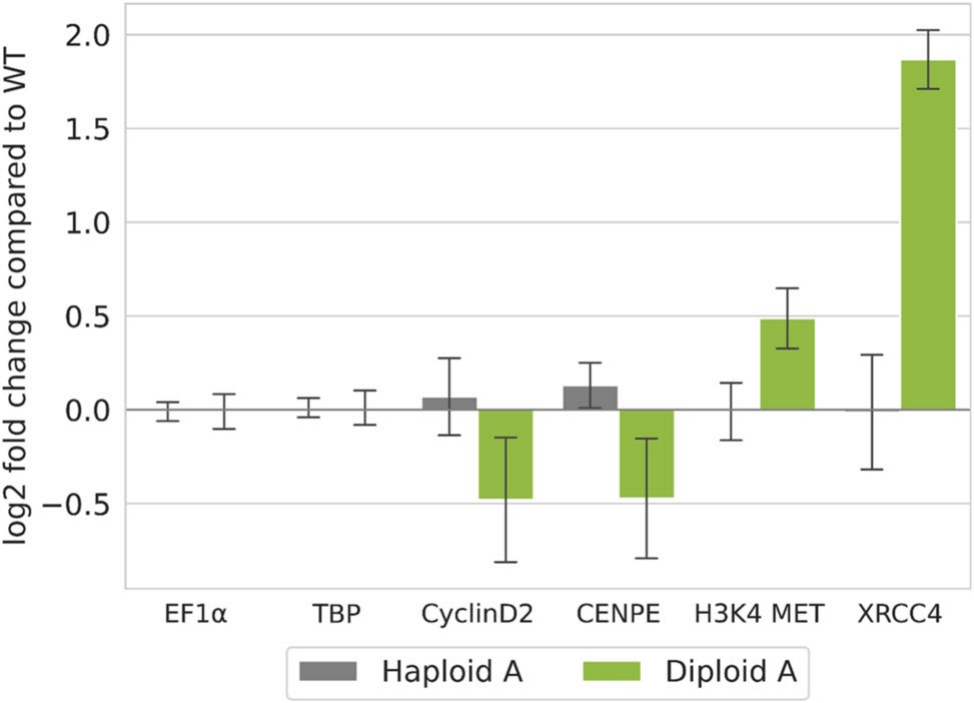
XRCC4 is upregulated in protonemata of the diploid Physcomitrella line. Relative transcript abundance in haploid and diploid Physcomitrella lines compared to WT as measured by real-time qRT-PCR. Normalized relative quantities were computed for each of three biological replicates according to Hellemans et al. (2007). Depicted is the mean log2 fold change over the replicates, error bars represent the standard deviations. EF1α and TBP are shown as reference for ploidy-independent gene expression

To experimentally validate these DEGs*,* transcript abundances in three lines (WT, Haploid A, Diploid A) were quantified via real-time qRT-PCR. RNA was isolated from protonema in biological and technical triplicates. In the qRT-PCR analysis, an upregulation of H3K4-Methyltransferase and cyclin D2 as observed in the first two-factor analysis (Diploid A versus WT and Haploid A including all protoplast and protonema samples) as well as of CENPE as observed in the first and second two-factor analyses (Diploid A versus WT including all protoplast samples) was not supported in diploid protonema (Fig. 4). In contrast, a ploidy-dependent expression of XRCC4 in protonemal tissue was validated as noticeably different from WT control in the diploid line by qRT-PCR with a 1.86 ± 0.16 log2 fold increase in transcript abundances for Diploid A. Next, we checked XRCC4 expression in natural developmental stages of WT Physcomitrella in publicly available datasets (Fernandez‐Pozo et al. 2020). We selected the four datasets that contain values for gene expression in the sporophyte from the ecotypes Gransden and Reute: RNAseq developmental stages v3.3 (Gransden and Reute), CombiMatrix Developmental stages gmv1.2 (Gransden and Reute), NimbleGen Developmental and Mycorrhiza gmv1.6 (Reute; mycorrhiza exudate and heat treated samples were not considered), and NimbleGen gmv1.6 (Gransden). The first three datasets contained sporophytic data only from the Reute ecotype, whereas in the last it was from the Gransden ecotype. In all datasets, we observed a tendency for high XRCC4 expression in the natural diploid sporophytic developmental stages (Supplementary Figure S6). The highest XRCC4 expression was always in one of the sporophytic stages even though XRCC4 expression was in some sporophytic developmental stages lower or comparable to other haploid stages. This was the case in the NimbleGen dataset, where only the brown sporophytes of the Gransden ecotype showed especially high XRCC4 expression levels while in the earlier green sporophytic stages XRCC4 expression was not enhanced compared to archegonia and spores (Supplementary Figure S6c). In contrast, in the NimbleGen Developmental and Mycorrhiza dataset and the RNAseq developmental stages dataset XRCC4 expression was highest in the green sporophyte of the Reute ecotype but the level in brown sporophytes was lower and comparable to that in juvenile gametophores (Supplementary Figure S6b, d). Similar to XRCC4, the highest expression of cyclin D2 in all four datasets was in one of the sporophytic stages (Supplementary Figure S7a-d). However, cyclin D2 was also strongly expressed in other developmental stages, for example in the spores (Gransden) in the RNAseq dataset (Supplementary Figure S7d). For CENPE and the H3K4-Methyltransferase, the expression in the sporophytes was not considerably higher or even much lower than in the other developmental stages (Supplementary Figs. S8, S9). Only the embryo data of the NimbleGen dataset from Ortiz-Ramírez et al. (2016) showed a quite high expression of both genes, but the expression drastically decreased during subsequent development (Supplementary Figs. S8c, S9c).

### GT rate is reduced in diploid Physcomitrella cells

**Fig. 5:**
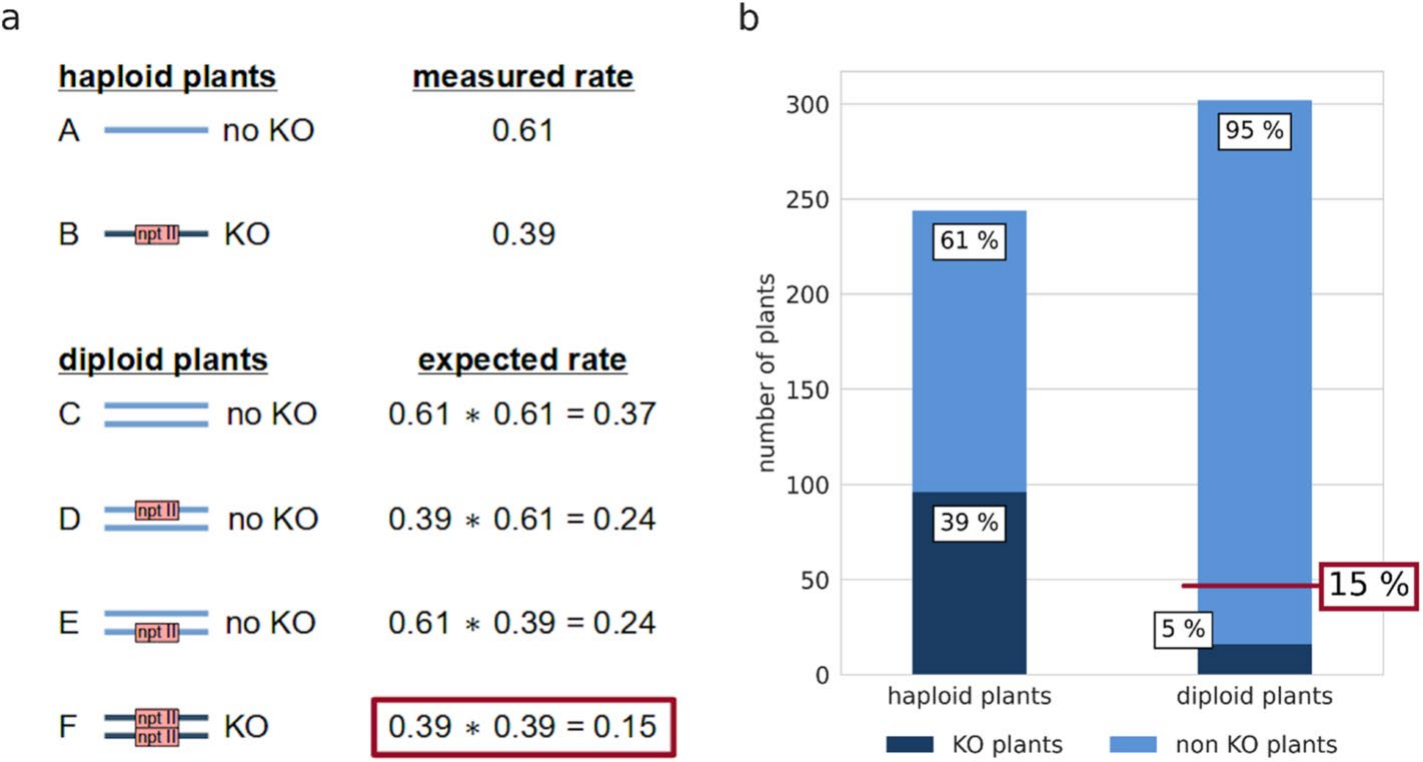
Diploid Physcomitrella cells show significantly lower GT rates. **a**: Estimation of the expected GT rate in diploid Physcomitrella plants computed from the measured GT rate in haploid plants. Shown are the observed rates of untransformed (*A*) and transformed (*B*) haploid plants as well as the expected rates of diploid plants having no integration of the cDNA construct in both chromosomes (*C*), having the cDNA construct integrated in only one chromosome (*D* and *F*) and having a full knock-out (KO) of the target locus in both chromosomes (*F*). The genomic loci are represented as solid lines and integration of a cDNA-construct containing the npt II cassette as selection marker is indicated. **b**: Comparison of GT rates in haploid and diploid Physcomitrella plants as determined via PCR analysis. For haploid plants, 96 out of 244 transformants are targeted KOs while for diploid plants, only 16 out of 302 transformants are targeted KOs on both chromosomes. The expected value of targeted KOs under the assumption of equal GT frequency for haploid and diploid plants is 15% (marked in red)

Next, we determined the ratios of GT via HR to illegitimate integration via NHEJ in two haploids (Haploid A, Haploid B) and three diploids (Diploid A, Diploid B, Diploid C). These lines were transformed with a KO construct containing a 1920 bp cDNA fragment of the cold-responsive gene Pp3c21_180V3, encoding sphingolipid fatty acid desaturase (PpSFD) (Beike et al. 2015; Hohe et al. 2004; Resemann et al. 2021). The GT rate was defined as the number of KO plants divided by the total number of transformants – the latter characterized by survival on selection medium and the presence of the selection marker (npt II; verified via PCR analysis). All transformants were tested by flow cytometry to determine their ploidy, resulting in the identification of 244 haploid and 302 diploid transformants (Fig. 5b). All 546 plants were tested with primers JMLKO25L and JMLKO25R flanking the insertion site of the KO cassette (Supplementary Table T1; Supplementary Figure S2). This approach screens for the presence or absence of the intact WT locus of the target gene. KO plants were characterised by the interruption of the WT locus for haploid plants and by interruption at both chromosomes for diploid plants (Fig. 5a). This procedure showed a disruption of the WT locus in 96 haploid KO plants, corresponding to a GT rate of 0.39. In diploids, successful GT requires the knockout in both chromosomes and hence the expected GT rate would be the square of the GT rate in haploid plants: 0.39 ∗ 0.39 = 0.15. However, for the diploid lines only 16 plants had a disrupted WT locus on both chromosomes, corresponding to a GT rate of 0.05. This value is significantly lower than the predicted rate of 0.15 (Fisher’s exact test, *p* < 0.001), revealing that the GT frequency is significantly reduced in diploids. Transformants with successful GT were checked for correct 5' and 3' integration of the construct into the Physcomitrella genome via PCR. 51of the 96 haploid plants showed proper integration patterns on both ends (5' and 3'), while it was 4 out of 16 in diploids. These different levels of PCR analyses enabled us to exclude illegitimate integrations and unstable transformants generated by "AltNHEJ" (Kamisugi et al. 2006) from our analysis. Notably, not only the GT frequency was strongly reduced in the diploids, but also the rate of proper gene replacement compared to “one-end-targeting”.

## Discussion

In this study, we investigated if a WGD, induced by protoplast fusion, leads to qualitative alterations in gene expression. Such qualitative changes might contribute not only to phenotypical changes (Egener et al. 2002; Schween et al. 2005a) but also be relevant for diverging GT success rates between angiosperms and moss. Physcomitrella is a particularly suitable model organism for this purpose because it grows in a life cycle where the haploid gametophytic stage is dominant and consequently diploidized Physcomitrella plants have, contrary to wild-type diploid species, two identical copies of each chromosome (autopolyploids). Thereby, we can exclude that transcriptomic differences arise from natural variability in the pair of homologous chromosomes, as is the case in allopolyploid hybrids. Here, we designed a multi-layer study using at first a fine-grained microarray time-series from samples taken during the 72 h regeneration of transfected haploid WT protoplasts, to investigate how protoplast recovery affects gene expression in the time period when GT is expected to take place. This first time-series was followed by a second microarray experiment and the more sensitive SuperSAGE technology to identify general transcriptomic differences between haploids and diploids at three time-points during protoplast regeneration. Finally, we set up two-factor analyses to test for expression changes caused by ploidy.

For protoplasts, the removal of the cell wall and the isolation of the single cell exposing it to a new environment represent massive, highly stressful interventions. During regeneration, Physcomitrella protoplasts partially reprogram into stem cells without the addition of plant hormones (Schween et al. 2003b). Xiao et al. (2012) identified transcriptomic changes during reprogramming via digital gene expression tag profiling (DGEP) at three time-points after protoplast isolation: 24 h versus 0 h, 48 h versus 24 h and 72 h versus 48 h. Here, we investigated the process via a microarray time series and concentrated on early events. Therefore, we sampled tissues during the first 24 h after transfection, using protoplast samples from 1 h, 4 h, 6 h, 24 h, and additionally from 72 h after transfection, and compared them to freshly isolated (0 h) protoplasts.

At 1 h, protoplasts had not yet adapted gene transcription levels, as we found no DEGs compared to freshly isolated protoplasts. In contrast, at 4 h cells had already started reprogramming, as evidenced by more than 200 DEGs. These encode, among others, enzymes involved in l-phenylalanine catabolism or in aromatic amino acid family catabolism. In our study, phenylalanine ammonia lyase, an enzyme that catalyzes the first step of the phenylalanine catabolic process (Hyun et al. 2011), was differentially expressed at 4 h and at 72 h. This gene is regulated in reaction to biotic and abiotic stresses and is the entry-point enzyme of the phenylpropanoid pathway, thereby supplying the basis for the synthesis of many downstream products like flavonoids. These compounds function as UV-filter, antioxidants and in drought resistance (Kumar et al. 2018). Moreover, flavones are extracellular signals for the root microbiome, especially under nitrogen deprivation (Yu et al. 2021). Further at 4 h, DEGs with the GO term glycolytic process were enriched (Fig. 1c), including the glycolysis key player glyceraldehyde-3-phosphate dehydrogenase (Supplementary Excel sheet 1). This enzyme is inhibited under oxidative stress by a redox modification of a cysteine residue. Additionally, there is evidence that in plants this protein fulfils non-metabolic functions under stress conditions supported by redox modifications of the enzyme (Gurrieri et al. 2021; Schneider et al. 2018; Wood et al. 2004). Nearly the whole set of genes that were differentially expressed at 6 h also belongs to the group of genes that plays a role in protoplast regeneration at 4 h (Fig. 1b).

A second peak became apparent at 24 h, resulting in the highest number of DEGs. At this time, an enrichment of DEGs with a function in aminomethyltransferase activity was apparent, which is in accordance with Xiao et al. (2012). In contrast to those authors, we did not detect enriched GO terms considering protein folding or reaction processes to the environment like response to salt stress, cold or heat at 24 h. In accordance to Xiao et al. (2012), however, we observed a significant enrichment of DEGs acting in photosynthesis at 24 h. In Xiao et al. (2012) most of the photosynthesis-related DEGs were downregulated at this time. A reduction of photosynthesis is a response to abiotic and biotic stresses and a trade-off in the distribution of means between growth and defense (Attaran et al. 2014; Cohen and Leach, 2019). In Xiao et al. (2012) the expression of many photosynthesis-related genes increased again at 48 h, probably to supply the necessary energy for the protoplasts that re-entered the cell cycle.

Accordingly, at 72 h the DEGs identified here were less dominated by genes associated with photosynthesis, indicating that the cellular energy management returned to normal. At this time, most protoplasts have undergone the first cell division (Xiao et al. 2012), arriving at a new stage with new transcriptomic requirements. Accordingly, we observed more than 200 new DEGs indicating the re-initiation of various physiological and cell-cycle dependent processes.

Overall, protoplast regeneration requires the regulation of important cellular processes that change gradually over time, and only 18 genes were always differentially expressed between 4 and 72 h. Among them is a gene of the GRAS family encoding a homologue to the gibberellin regulatory protein RGL1 of Arabidopsis. In Physcomitrella, the gibberellin precursor ent-kaurene plays a role in developmental regulation (Hayashi et al. 2010) while the gibberellin signaling pathway as it exists in angiosperms is not present in Physcomitrella (Vandenbussche et al. 2007). Another gene that was differentially expressed at all time-points encodes a catalase. Catalases degrade H_2_O_2_, and are indicators for oxidative stress (Smirnoff and Arnaud 2019; Yong et al. 2017). Upregulation of various types of stress-response genes during cell wall regeneration of protoplasts is reported also for cotton and rice (Sharma et al. 2011; Yang et al. 2008).

Subsequently, we generated more detailed transcriptomic data from several haploid and diploid lines using microarray and SuperSAGE technology. We analysed freshly isolated protoplasts and protoplasts at 4 h and 24 h after transfection, respectively. The DEGs of the haploids at 24 h compared to freshly isolated protoplasts covered 69.71% of the 1195 DEGs at this time-point in regenerating haploid Physcomitrella protoplast from Xiao et al. (2012). In addition, we identified 5898 DEGs, presumably due to different search criteria: We used different detection methods (microarray, SuperSAGE), two different haploid lines, and different filter criteria for DEGs (|log2 fold change|> 1 and *p* < 0.001 for microarray data and GFOLD(0.01) value <  − 1 or > 1 for SuperSAGE data in our studies; |log2 fold change|≥ 2, *p* ≤ 0.01 and FDR < 0.01 in Xiao et al. (2012)]. Examples of DEGs with high fold changes that were not common between our study and that from Xiao et al. (2012) are a cytochrome P450 in Xiao et al. (2012) and an ap2 erf domain-containing transcription factor in our data, both playing roles in development and stress (Gu et al. 2017; Hiss et al. 2014; Xu et al. 2015).

Next, we compared haploid and diploid protoplasts during reprogramming and regeneration at 0 h, 4 h and 24 h. A PCA of the SuperSAGE libraries revealed that a WGD had a much weaker effect on gene expression than the generation of protoplasts itself, or their subsequent regeneration. Indeed, haploids and diploids followed similar time-steps of regeneration. In all samples of diploids and haploids more genes were differentially expressed at 24 h than at 4 h (Supplementary Table T6). Independent of ploidy, photosynthesis-related genes were enriched in the DEGs of the SuperSAGE data at 4 h and 24 h (Supplementary Excel sheet 2). Independent of ploidy, the important role for DNA–DSB repair in regenerating protoplasts was reflected by differential expression of genes attributed to both types of repair pathways: genes acting in HR and genes relevant for NHEJ, most of them upregulated. For example, in haploids and diploids we detected at different time-points an upregulation of the HR key player Rad50 as well as a synchronous upregulation over time of the NHEJ key player XRCC4.

However, when directly comparing haploid and diploid protoplasts at 0 h, 4 h and 24 h, we discovered several ploidy-dependent DEGs. Surprisingly, most of them were only differentially expressed in diploids versus haploids at one of the time-points, hinting towards time-point-specific variations in DNA–DSB repair in diploids (Table 2, Fig. 3). According to our two-factor analyses, 3.7% of the Physcomitrella protein-encoding genes were DEGs in response to the WGD, but mostly with only a moderate change in expression. This clearly indicates a ploidy-dependent gene expression pattern in Physcomitrella protoplasts. The reported values of DEGs from diverse plant species after an artificial autopolyploidization vary strongly. For example, the amount of DEGs in diploid versus tetraploid *Paspalum notatum* is only 0.49%, whereas in *Zea mays* the reaction is stronger with over 26% DEGs (Spoelhof et al. 2017).

The transcriptomic differences between diploid and haploid plants suggest that diploidization might have an influence on the phenotype. Several studies addressed this issue in Physcomitrella*.* One of them considered 500 mock-transformed haploid and diploid plants (Schween et al. 2005a), while two others included data from 16,203 (Egener et al. 2002) or 73,329 (Schween et al. 2005b) haploid and polyploid transformants. In these studies only less than half of the polyploid plants showed normal growth on (minimal) Knop medium. In contrast, for the vast majority of the haploids, as well as in Schulte et al. (2006), who investigated 51,180 haploid knock-out mutants, growth on Knop medium was normal. For example, in Schween et al. (2005a) more than 90% of the haploids grew normally on Knop medium but only 20% of the diploids. Besides, a weak correlation of 0.55 between ploidy and growth on Knop medium was reported. However, considering the growth on Knop medium, the diploids analysed in our study were specifically selected not to behave significantly different from the haploids. Another feature that correlated with the ploidy level in Schween et al. (2005a) was the rate of coverage with gametophores that was comparable to WT in 74.9% of the haploids but only in 7.6% of the diploids. Furthermore, the leaf shape correlated with ploidy level; e.g., 25% of the diploids had a double leaf tip compared to only 0.1% of the haploids. Multiple phenotypic deviations were a feature correlating with ploidy that happened in about a quarter of the diploids but in none of the haploids. In total, much more haploid than diploid gametophores looked similar to the wild type with 93.1% and 26.3%, respectively. Other features apparently interlinked with the ploidy level as reported in Schween et al. (2005a) are the plant structure and the uniformity of leaves. We identified 9 ploidy-dependent DEGs with a |log2 fold change| in expression of more than 1.5 that are associated with developmental process or growth (Supplementary Table T10). These include genes associated with anatomical-structure development, leaf morphogenesis and regulation of meristem growth.

Next, we searched for ploidy-dependent DEGs associated with DNA–DSB repair, cell cycle regulation and DNA accessibility. Since HR occurs in the same period as protoplast regeneration, survival and regeneration of protoplasts are responsible for a strong “background noise” of DEGs, that might mask subtle transcriptomic responses. We detected an upregulation of HR- and NHEJ-related genes at some time-points. Only few HR-relevant genes were downregulated in diploids, for example DNA polymerase I. Further, the gene encoding Polymerase Q (POLQ) that is unfavourable for GT via HR in Physcomitrella (Mara et al. 2019), was upregulated in the same cells. One especially interesting candidate was the gene encoding the Physcomitrella homologue of XRCC4, a key player in the NHEJ DNA–DSB repair pathway in mammals (Chang et al. 2017), which is also upregulated in Physcomitrella after bleomycin-induced DNA damage in haploids (Kamisugi et al. 2016). Here, we detected an upregulation of XRCC4 not only in diploid versus haploid protoplasts at 4 h and 24 h, but it was also the DNA–DSB repair-relevant gene with the highest expression change in the two-factor analysis of the SuperSAGE data (Supplementary Excel sheet 1). Quantitative real-time PCR validated that the XRCC4 transcript level is ploidy-dependent with a much higher transcript abundance in diploids than in haploids (Fig. 4). An investigation of the XRCC4 expression profile during the Physcomitrella life cycle in publicly available transcript data (Fernandez‐Pozo et al. 2020) revealed a tendency to higher XRCC4 transcript levels in the natural diploid life stage, the sporophyte, than in haploid protonema and protoplasts (Supplementary Figure S6a–d). These findings suggest that the choice of the DNA–DSB repair pathway is most likely dependent on the ploidy level, not only in plants before and after a WGD, but also in the natural haploid and diploid developmental stages of WT Physcomitrella. The existence of such an interdependency between ploidy level and the repair pathway choice was reported for haploid and diploid yeast cells under DNA replication stress (Li and Tye 2011).

After having identified DEGs relevant for the repair of DNA–DSBs, and thus potentially for the choice between HR and NHEJ, in haploid versus diploid protoplasts, we analysed GT frequencies between both cell types. The GT construct pRKO25.2 (Hohe et al. 2004) was utilized to transform two haploid and three WGD lines of Physcomitrella. Surprisingly, transformation of diploid protoplasts yielded only 5% true knockouts instead of the theoretically expected 15% (Fig. 5), revealing a significant suppression of GT after WGD. Hohe et al. (2004) compared GT rates of different single cDNA constructs with mixes of 5 or 10 cDNAs and found no difference between single and mixed cDNA, indicating that for our transformation protocol the uptake of cDNA is not a limiting factor for HR. Martin et al. (2009) showed that the production of double FtsZ-mutants can be as effective as the production of single mutants, confirming that the amount of cDNA during transformation is sufficient for several loci at the same time. Hence, in diploid Physcomitrella lines increased expression of the gene encoding XRCC4 correlates with a suppression of GT and thereby, the NHEJ pathway gains in significance over HR, the main DNA–DSB repair mechanism of the haploid-dominant moss (Kamisugi et al. 2006). We interpret high NHEJ rates in diploids as a reduced selective pressure for accurate DNA repair due to the additional information back-up available in form of a second set of chromosomes. Elevated NHEJ rates in diploids support the hypothesis that the haploid phase of Physcomitrella is interlinked with high integration rates of transgenes via HR (Schaefer and Zrÿd 1997). Yet, ploidy is unlikely the sole factor that determines GT rates in plants for several reasons: (i) GT frequencies of seed plants did not increase with haploid tissues (Mengiste and Paszkowski 1999), (ii) GT in other haploid species like *Volvox* is not as efficient as in Physcomitrella (Reski 1998b), and (iii) the GT rate we measured in diploid Physcomitrella plants is still a multiple factor higher than GT rates observed in polyploid angiosperms. Another factor potentially contributing to the GT efficiency in Physcomitrella is the G2/M-phase arrest of the protonema tissue used for transformation. This was, however, unchanged after WGD in our diploids.

As we analysed the transcriptomic responses in bulks of 300,000 protoplasts each, DEGs may have been masked by different transformation efficiencies or by the bulk of untransformed protoplasts. However, we did not observe different transformation efficiencies between haploid and diploid protoplasts based on the highly standardized procedures developed by us (Hohe et al. 2004). Single-cell transcriptomic studies are gaining popularity (Cole et al. 2021) but are still in their infancy in Physcomitrella (Kubo et al. 2019) and thus not highly standardized for a series of quantitative studies we performed here. The differences in gene expression between haploids and diploids having an identical, albeit duplicated, genome might be to some extent caused by ploidy-dependent epigenetic regulation of the transcriptome. Epigenetic regulation of chromatin accessibility is partially mediated via chromatin marks. Xiao et al. (2012) showed that various methyltransferases are DEGs during protoplast regeneration in Physcomitrella. This may indicate an important mechanism for epigenetic regulation of DNA repair pathways. Indeed, epigenetic alterations (Wolffe and Matzke 1999) as well as the adaption of gene-regulatory networks and direct changes in the genome structure, among others by an altered transposable element activity or homologous and non-homologous recombination (Adams and Wendel 2005; del Pozo and Ramirez-Parra 2015; Liu and Wendel 2003; Otto 2007), already happen in the first generations very shortly after a WGD. They are reactions to challenges arising in newly formed polyploids, like genetic instability (Soltis et al. 2015), an increased demand of energy and a higher number of chromosomes to deal with during mitosis (del Pozo and Ramirez-Parra 2015; Doyle et al. 2008).

With the creation of artificial diploid Physcomitrella plants we have imitated a WGD event, which is an important driving force of evolution that happened several times over the past 200 million years in land plants (Renny-Byfield and Wendel 2014; Soltis and Soltis 2016; van de Peer et al. 2017), including Physcomitrella (Lang et al. 2018). Our studies provide an insight into the adaption of gene expression following a WGD. Such findings might help to retrace how autopolyploids established during evolution. Additionally, we are one step closer to unmasking the mysteries surrounding GT in plants by further elucidating the regulation of DNA repair mechanisms. Understanding the mechanism of HR is the basis for transferring the technique and efficiency to create genetically modified organisms via GT from Physcomitrella to other plant species (Collonnier et al. 2017). The biological relevance of DEGs described here will be analysed in loss-of-function moss mutants generated by GT in forthcoming studies.

## Supplementary Information

Below is the link to the electronic supplementary material.

Supplementary file1 (DOCX 1408 KB)

Supplementary file2 (XLSX 4704 KB)

Supplementary file3 (XLSX 674 KB)

## Declarations

### Data availability

All data are included in this manuscript and its supplement. All plant material has been deposited in the International Moss Stock Center (IMSC). The respective IMSC numbers are provided in the manuscript.
