# Supplementary material for: Autopolyploidization affects transcript patterns and gene targeting frequencies in Physcomitrella
Source: Plant Cell Rep. 2021 Oct 12;41(1):153–73. doi: 10.1007/s00299-021-02794-2 (PMC8803787; doi:10.1007/s00299-021-02794-2)

**Supplementary Information**

**Autopolyploidization affects transcript patterns and gene targeting frequencies in Physcomitrella**

Christine Rempfer^a,b^, Gertrud Wiedemann^a,e^, Gabriele Schween^a,f^, Klaus L. Kerres^a^, Jan M. Lucht^a,g^, Ralf Horres^c^, Eva L. Decker^a^, Ralf Reski^a,b,d,*^

^a^Plant Biotechnology, Faculty of Biology, University of Freiburg, Schaenzlestr. 1, 79104 Freiburg, Germany

^b^Spemann Graduate School of Biology and Medicine (SGBM), University of Freiburg, 79104 Freiburg, Germany

^c^GenXPro GmbH, Altenhöferallee 3, 60438 Frankfurt am Main, Germany;

^d^Signalling Research Centres BIOSS and CIBSS, Schaenzlestr. 18, 79104 Freiburg, Germany

Current Addresses:

^e^Department of Hematology and Central Hematology Laboratory, Inselspital, Bern University Hospital, University of Bern, 3010 Bern, Switzerland

^f^Corteva Agriscience, Pioneer Hi-Bred Northern Europe, Münstertäler Strasse 26, 79427 Eschbach, Germany

^g^scienceindustries, Nordstrasse 15, 8006 Zurich, Switzerland

ORCID IDs: 0000-0002-0672-3897 (CR), 0000-0002-2259-1011 (GW), 0000-0002-4722-1244 (JML), 0000-0002-9151-1361 (ELD), 0000-0002-5496-6711 (RR)

*Corresponding author: RR ralf.reski@biologie.uni-freiburg.de

**Supplementary Figure S1** Flow cytometric analyses of haploid and diploid Physcomitrella lines. For haploid lines (left), peaks at a fluorescence intensity of about 200 represent cells in the G2 phase whereas cells in the G1 phase have a fluorescence intensity of about 100. For diploid lines (right), the peaks at a fluorescence intensity of about 400 represent cells in the G2 phase and cells in the G1 phase have a fluorescence intensity of about 200. The FCM images were published in Schween et al. (2005a).


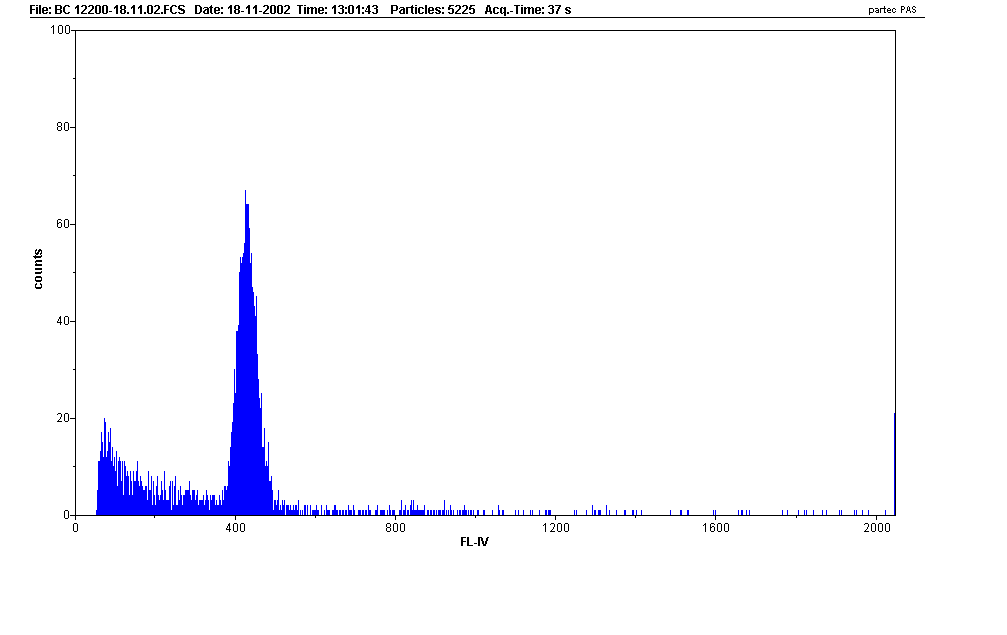

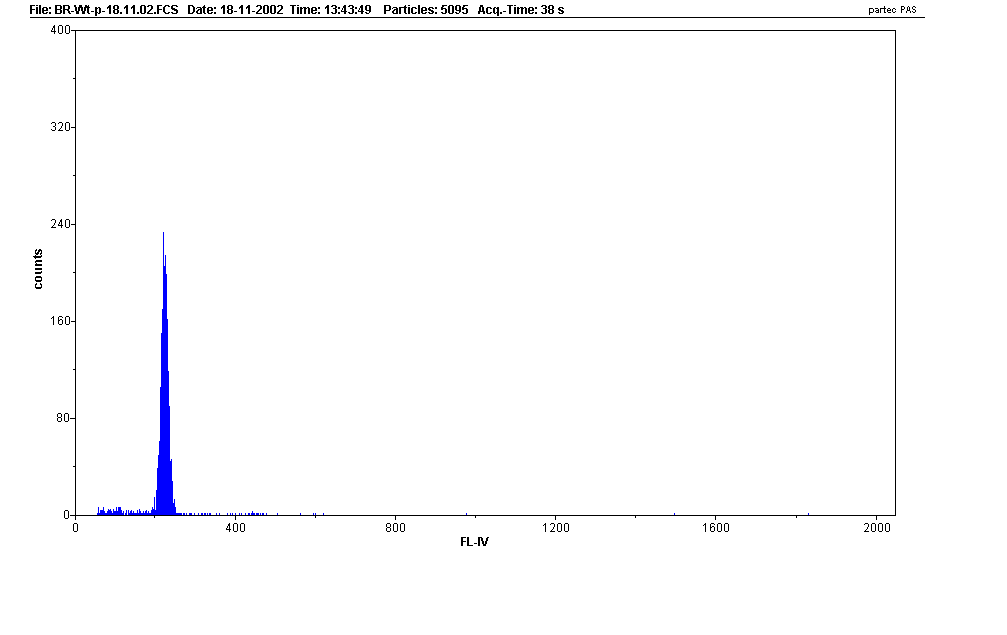
 WT Diploid A

**
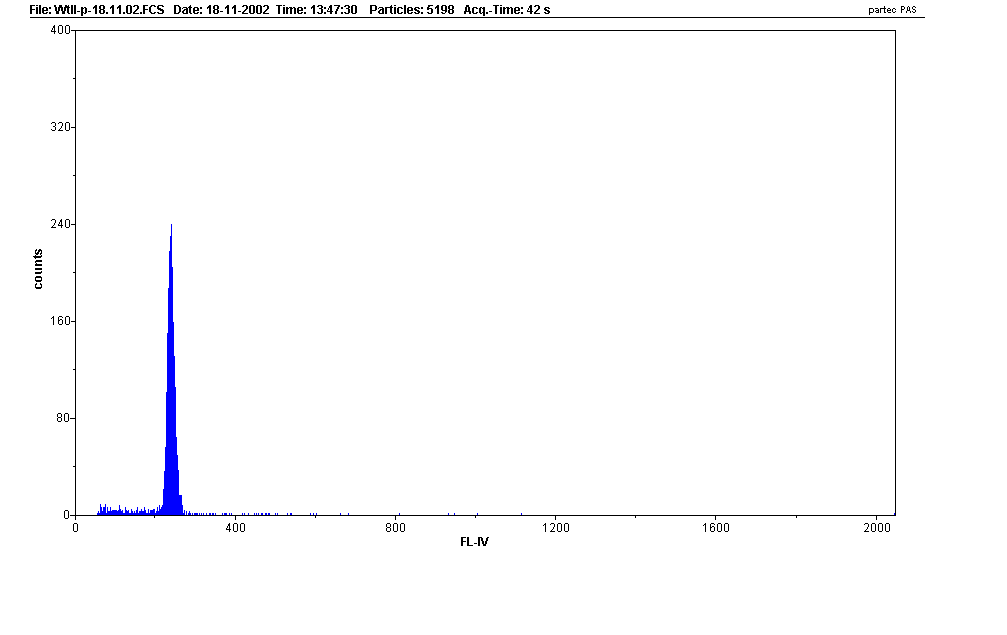

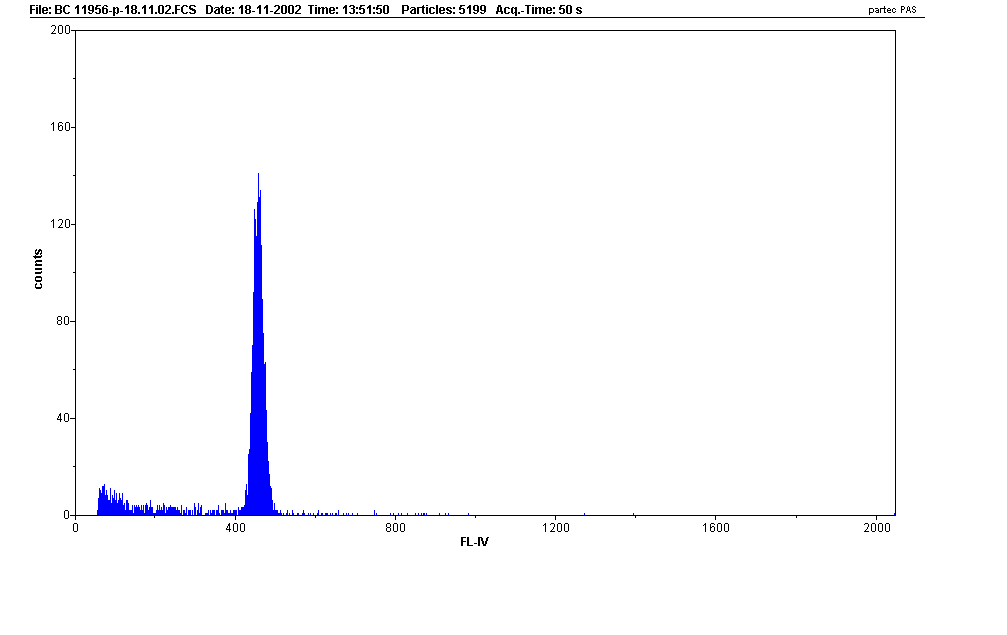
** Haploid A Diploid B

**
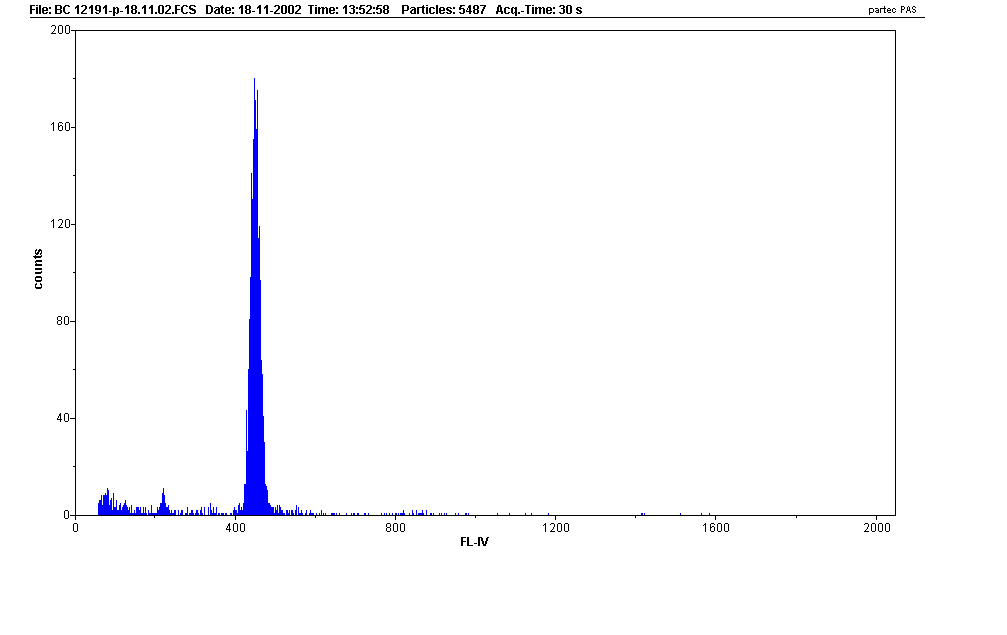
** Haploid B Diploid C **
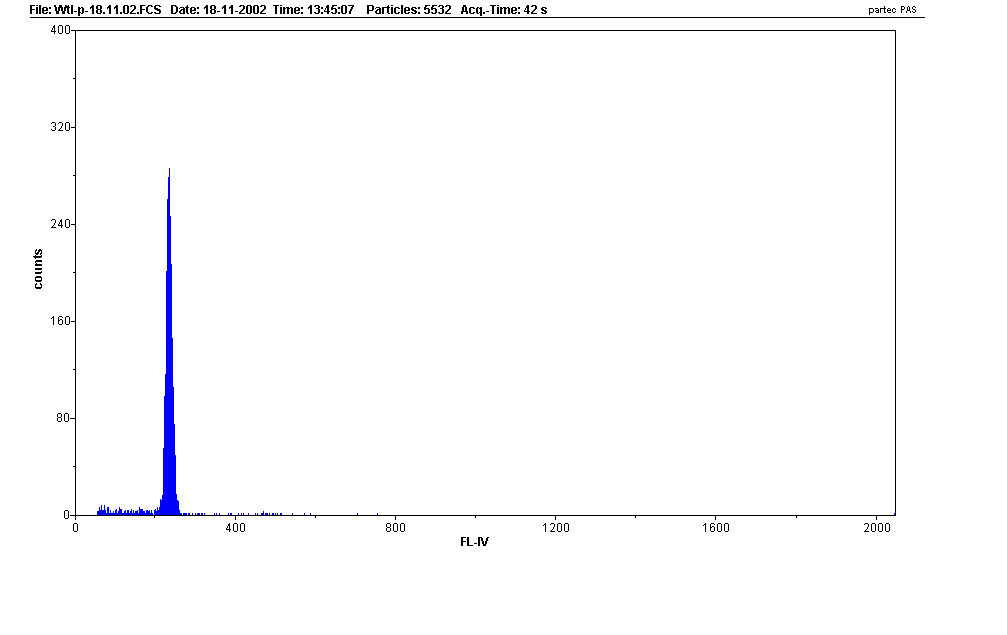
**

Supplementary Table T1 List of primers used for PCR screening of transformed Physcomitrella plants and for quantitative real-time PCR (qRT-PCR) of Physcomitrella cDNA.

| **Primer name** | **Sequence** | **Function** |
| --- | --- | --- |
| **npt2cdc1-L** | 5'-TGA ATG AAC TGC AGG ACG AG-3' | detection of npt II-cassette |
| **npt2cdc1-R** | 5'-AGC CAA CGC TAT GTC CTG AT-3' | detection of npt II-cassette |
| **JMLKO25L** | 5'-CTC CAG TTG TCG GAG AAG GCG-3' | amplification of (endogenous) wild-type band |
| **JMLKO25R** | 5'-ACT CTG GAG CAG CTT CAT GGC-3' | amplification of (endogenous) wild-type band |
| **JMLKO25-L3** | 5'- CGC CTC ACG GTT AAG GCA T-3' | 5' integration screening (endogenous flanking region inward) |
| **JMLK2-R5** | 5'-AAC GTG ACT CCC TTA ATT CTC CGC-3' | 5' integration screening (npt II-cassette outward) |
| **JMLK2-F3** | 5'-TGT TAC TAG ATC GGG CCT CCT GTC A-3' | 3' integration screening (npt II-cassette outward) |
| **JMLKO25-L4** | 5'-ACA GCG AAT GCA GAG AAA AGA GCC-3' | 3' integration screening (endogenous flanking region inward) |
| **CEN_Ex5_F** | 5'-GGA AAT CGT TGA CAG TGT TGA A-3' | Pp3c22_20430V3 forward primer for qPCR |
| **CEN_Ex6_R** | 5'-TTT CTC CGA AAT GAC GTT GA-3' | Pp3c22_20430V3 reverse primer for qPCR |
| **CYCD2_Ex3_F** | 5'-GAG GTT CCG ATT CTT CTG GAC-3' | Pp3c9_8300V3 forward primer for qRT-PCR |
| **CYCD2_Ex4_R** | 5'-AGT GTG AGC TTC AAA GAT GTG C-3' | Pp3c9_8300V3 reverse primer for qRT-PCR |
| **MET_Ex1_F** | 5'-ACT CCT TAC ACG GAC GAG GA-3' | Pp3c4_16880V3 forward primer for qRT-PCR |
| **MET_Ex2_R** | 5'-ACG ACA ACT CCC TTG CAG AT-3' | Pp3c4_16880V3 reverse primer for qRT-PCR |
| **XRCC4_41F** | 5'-GGC TTG GAC GTT TGA AGG-3' | Pp3c1_38430V3 forward primer for qRT-PCR |
| **XRCC4_41R** | 5'-CCC AGC TCA CAT TCT TGT CC-3' | Pp3c1_38430V3 reverse primer for qRT-PCR |
| **EF1a_qf** | 5'-CGA CGC CCC TGG ACA TC-3' | Pp3c2_10310V3 forward primer for qRT-PCR |
| **EF1a_qr** | 5'-CCT GCG AGG TTC CCG TAA-3' | Pp3c2_10310V3 reverse primer for qRT-PCR |
| **C45_tg2F** | 5'-ACG CAC CGG CAT CGT-3' | Pp3c13_2360V3 forward primer for qRT-PCR |
| **C45_tg2R** | 5'-TGC TTG TTC ATC ACG ACA CCA A-3' | Pp3c13_2360V3 reverse primer for qRT-PCR |
| **Tata_qf** | 5'-GAT CTA GCT ATA AGC CTG ATC TAC CG-3' | Pp3c12_4720V3 forward primer for qRT-PCR |
| **Tata_qr** | 5'-CAG GAG CAG GGA GAG ATT TG-3' | Pp3c12_4720V3 reverse primer for qRT-PCR |

Supplementary Figure S2 Schematic overview of knock-out construct pRKO25.2 integrated in the Physcomitrella genome. The location of primers used for PCR analysis of wild-type locus, 5' integration and 3' integration is shown.


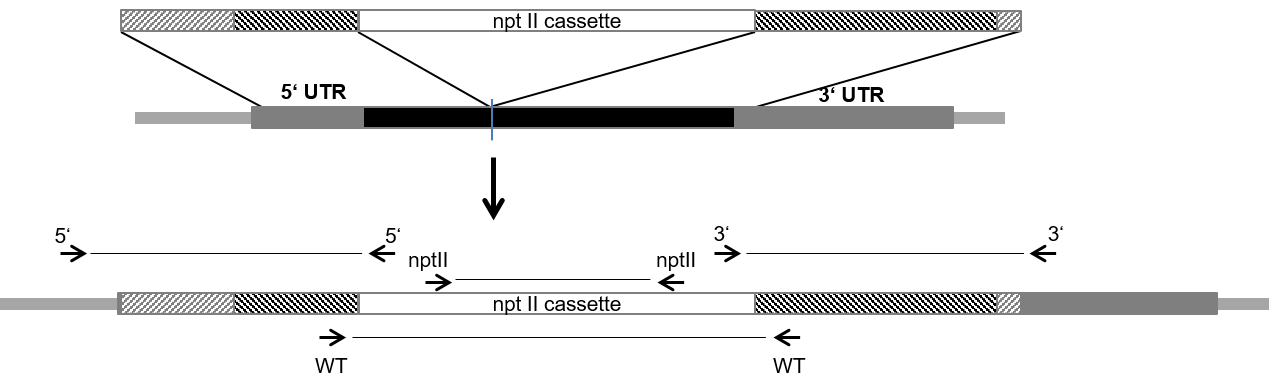


**Supplementary Table T2** Overview of the analyses performed with different Physcomitrella lines and sample sources. Differentially expressed genes were computed for different factors (time; ploidy at a certain time-point after protoplast isolation and transfection; ploidy) using protonema (PN), freshly isolated protoplasts (0h) as well as protoplasts at 4h and 24h after transfection.

| **Factor** | **Lines** | **Comparisons** | | |
| --- | --- | --- | --- | --- |
|  |  | **First microarray** | **Second microarray** | **SuperSAGE** |
| **Time** | WT | 1h vs. 0h  4h vs. 0h  6h vs. 0h  24h vs. 0h  72h vs. 0h |  | 4h vs. 0h  24h vs. 0h |
| **Time** | Haploid A |  | 4h vs. 0h  24h vs. 0h  PN vs. 0h | 4h vs. 0h  24h vs. 0h  PN vs. 0h |
| **Time** | Diploid A |  | 4h vs. 0h  24h vs. 0h  PN vs. P0h | 4h vs. 0h  24h vs. 0h  PN vs. 0h |
| **Ploidy (time-point specific)** | Diploid A vs. Haploid A |  | 0h  4h  24h  PN | 0h  4h  24h  PN |
| **Ploidy (time-point specific)** | Diploid A, Diploid B vs. WT, Haploid A |  | 0h  4h  24h  PN |  |
| **Ploidy (time-point specific)** | Diploid A vs. WT |  |  | 0h  4h  24h |
| **Ploidy** | Diploid A vs. WT, Haploid A |  |  | Two-factor analysis of: 0h, 4h, 24h and PN |
| **Ploidy** | Diploid A vs. WT |  |  | Two-factor analysis of: 0h, 4h and 24h |

**Supplementary Table T3** Overview of SuperSAGE libraries used for analysis of differential gene expression. Libraries are constructed from 26 nt long reads as obtained by SuperSAGE technology and are mainly characterized by cell line and cell type of the material. Samples were taken from freshly isolated protoplasts (0h) and protoplasts at 4h and 24h after transfection. The libraries marked with * are mock transformants not exposed to foreign DNA. They were subjected to the whole transfection procedure but using water instead of the GT construct.

| **Line** | **Ploidy** | **Tissue** | **Replicates** |
| --- | --- | --- | --- |
| Diploid A | Diploid | protoplast 0h | 1 |
| Diploid A | Diploid | protoplast 4h | 1 |
| Diploid A | Diploid | protoplast 24h | 1 |
| Diploid A | Diploid | protonema | 2 |
| Haploid A | Haploid | protoplast 0h | 1 |
| Haploid A | Haploid | protoplast 4h | 1 |
| Haploid A | Haploid | protoplast 24h | 1 |
| Haploid A | Haploid | protonema | 2 |
| WT | Haploid | protoplast 0h | 1 |
| WT | Haploid | protoplast 4h | 1 |
| WT | Haploid | protoplast 4h | 2* |
| WT | Haploid | protoplast 24h | 1 |
| WT | Haploid | protoplast 24h | 2* |

The number of sequenced reads of the different libraries ranged from approximately 1.49 x 10^6^ to 24.21 x 10^6^ per library while the number of distinct sequences per library ranged from approximately 0.18 x 10^6^ to 1.46 x 10^6^. An average of 81.8% of the sequences was mapped to the V3.3 annotation of the Physcomitrella genome (Lang et al., 2018). On average 84.6% of the mapped reads resulted in a valid alignment as defined per featureCounts parameters (Supplementary Table T5). The majority of reads in the SuperSAGE data were highly abundant. More than half of the reads represented single sequences that were present more than 500 times on average per library while more than 10% of the reads belonged to sequences appearing over 10,000 times on average per library.

**Supplementary Table T4** Applied input parameters for the HISAT2 (v2.0.3, Kim et al., 2015) tool on the Galaxy platform (Freiburg Galaxy instance, http://galaxy.uni-freiburg.de, Afgan et al., 2016). The SuperSAGE reads were mapped to the V3.3 assembly of the Physcomitrella genome and known splice sites were provided in a gene annotation file in gff3 format.

| **Input Parameter** | **Value** |
| --- | --- |
| Input data format | Fastq |
| Single end or paired reads? | Single |
| Primary alignments | 50 |
| Alignment options | Defaults |
| Input options | Defaults |
| Scoring options: Function type | Constant |
| Scoring options: Constant term (y) | -2 |
| Scoring options: Coefficient (z) | 0 |
| Scoring options: Set match bonus | 2 |
| Scoring options: Maximum mismatch penalty | 6 |
| Scoring options: Minimum mismatch penalty | 2 |
| Scoring options: Ambiguous read penalty | 1 |
| Scoring options: Maximum soft-clipping penalty | 2 |
| Scoring options: Minimum soft-clipping penalty | 1 |
| Scoring options: Read gap open penalty | 5 |
| Scoring options: Read gap extend penalty | 3 |
| Scoring options: Reference gap open penalty | 5 |
| Scoring options: Reference gap extend penalty | 3 |
| Spliced alignment parameters: Penalty for canonical splice sites | 0 |
| Spliced alignment parameters: Penalty for non-canonical splice sites | 3 |
| Spliced alignment parameters: Penalty for long introns with canonical splice sites | Constant |
| Spliced alignment parameters: Constant term (y) | 0 |
| Spliced alignment parameters: Coefficient (z) | 0 |
| Spliced alignment parameters: Penalty for long introns with noncanonical splice sites | Constant |
| Spliced alignment parameters: Constant term (y) | -8 |
| Spliced alignment parameters: Coefficient (z) | 1 |
| Spliced alignment parameters :Minimum intron length | 20 |
| Spliced alignment parameters: Maximum intron length | 500000 |
| Spliced alignment parameters: Specify strand-specific information | Second Strand (F/FR) |
| Disable spliced alignment | False |
| Transcriptome assembly reporting | Use default reporting |
| Paired alignment parameters | Defaults |

**Supplementary Table T5** Applied input parameters for the featureCounts tool (v1.4.6.p5, Liao et al., 2014) on the Galaxy platform (Freiburg Galaxy instance, http://galaxy.uni-freiburg.de, Afgan et al., 2016). Count tables were constructed from the output of the HISAT2 (v2.0.3) mapping tool using the V3.3 Physcomitrella annotation files.

| **Input Parameter** | **Value** |
| --- | --- |
| Output format | Gene-ID “\t” read-count |
| featureCounts parameters: GFF feature type filter | Exon |
| featureCounts parameters: GFF gene identifier | gene_id |
| featureCounts parameters: Allow read to contribute to multiple features | False |
| featureCounts parameters: Strand specific protocol | Unstranded |
| featureCounts parameters: Count multi-mapping reads/fragments | True |
| featureCounts parameters: Minimum read quality | 0 |
| Paired-end reads options: Count fragments instead of reads | True |
| Paired-end reads options: Check paired-end distance | False |
| Paired-end reads options: Minimum fragment/template length | 50 |
| Paired-end reads options: Maximum fragment/template length | 600 |
| Paired-end reads options: only allow fragments with both reads aligned | False |
| Paired-end reads options: Exclude chimeric fragments | True |
| On feature level | False |

**Supplementary Table T6** Number and overlap of genes being upregulated or downregulated between protonema samples or protoplasts at 4h or 24h after transfection versus freshly isolated protoplasts (0h). DEGs were computed from microarray (M) and SuperSAGE (S) data of two haploid lines (WT, Haploid A) and a diploid line (Diploid A). DEGs from the microarray experiment were determined with the Expressionist Analyst Pro software and were filtered for |log2 fold change| > 1 and p < 0.001. The SuperSAGE data analysis was performed with GFOLD and DEGs were filtered for a GFOLD(0.01) value of < -1 or > 1.

| Line | 4h versus 0h protoplast | | | | 24h versus 0h protoplast | | | | Protonema versus protoplast (0h) | | | | Overlap 4h vs. 0h with 24h vs. 0h |
| --- | --- | --- | --- | --- | --- | --- | --- | --- | --- | --- | --- | --- | --- |
|  | total | up | down | down  [%] | total | up | down | down  [%] | total | up | down | down  [%] | total |
| WT (S) | 1148 | 444 | 704 | 61.32 | 4000 | 1167 | 2833 | 70.83 | - | - | - | - | 963 |
| Haploid A (S) | 3002 | 978 | 2024 | 67.42 | 3265 | 860 | 2405 | 73.66 | 740 | 74 | 666 | 90.00 | 1701 |
| Diploid A (S) | 1453 | 491 | 962 | 66.21 | 3949 | 2905 | 1044 | 26.44 | 545 | 3 | 542 | 99.45 | 1085 |
| Haploid A (M) | 262 | 48 | 214 | 81.67 | 2273 | 1365 | 908 | 39.95 | 1453 | 366 | 1087 | 74.81 | 162 |
| Diploid A (M) | 309 | 260 | 49 | 15.86 | 1894 | 1575 | 319 | 16.84 | 1674 | 958 | 716 | 42.77 | 223 |
| Combined (M, S) Haploid A | 3142 | 995 | 2147 | 68.33 | 4663 | 1879 | 2784 | 59.70 | 1696 | 424 | 1272 | 75.00 | 1925 |
| Combined (M, S) Haploid A and WT | 3823 | 1297 | 2526 | 66.07 | 6698 | 2451 | 4247 | 63.41 | 1696 | 424 | 1272 | 75.00 | 2774 |
| Combined (M, S) Diploid A | 1628 | 653 | 975 | 59.89 | 4922 | 3714 | 1208 | 24.54 | 1860 | 960 | 900 | 48.39 | 1246 |

## Supplementary Figure S3 Overlap of differentially expressed genes (DEGs) from microarray and SuperSAGE data. DEGs were computed from protonema samples (a, b), protoplasts 4h after transfection (c, d) and protoplasts 24h after transfection (d, e), respectively, versus freshly isolated (0h) protoplasts using two haploid lines (a, c, d) and one diploid line (b, d, e). Results from microarray data are depicted in blue and results from SuperSAGE data in yellow. For our identified DEGs from the haploid lines at 24h € the overlap to DEGs from 24h old versus freshly isolated protoplasts identified by Xiao et al. (2012) is shown (pink).

**
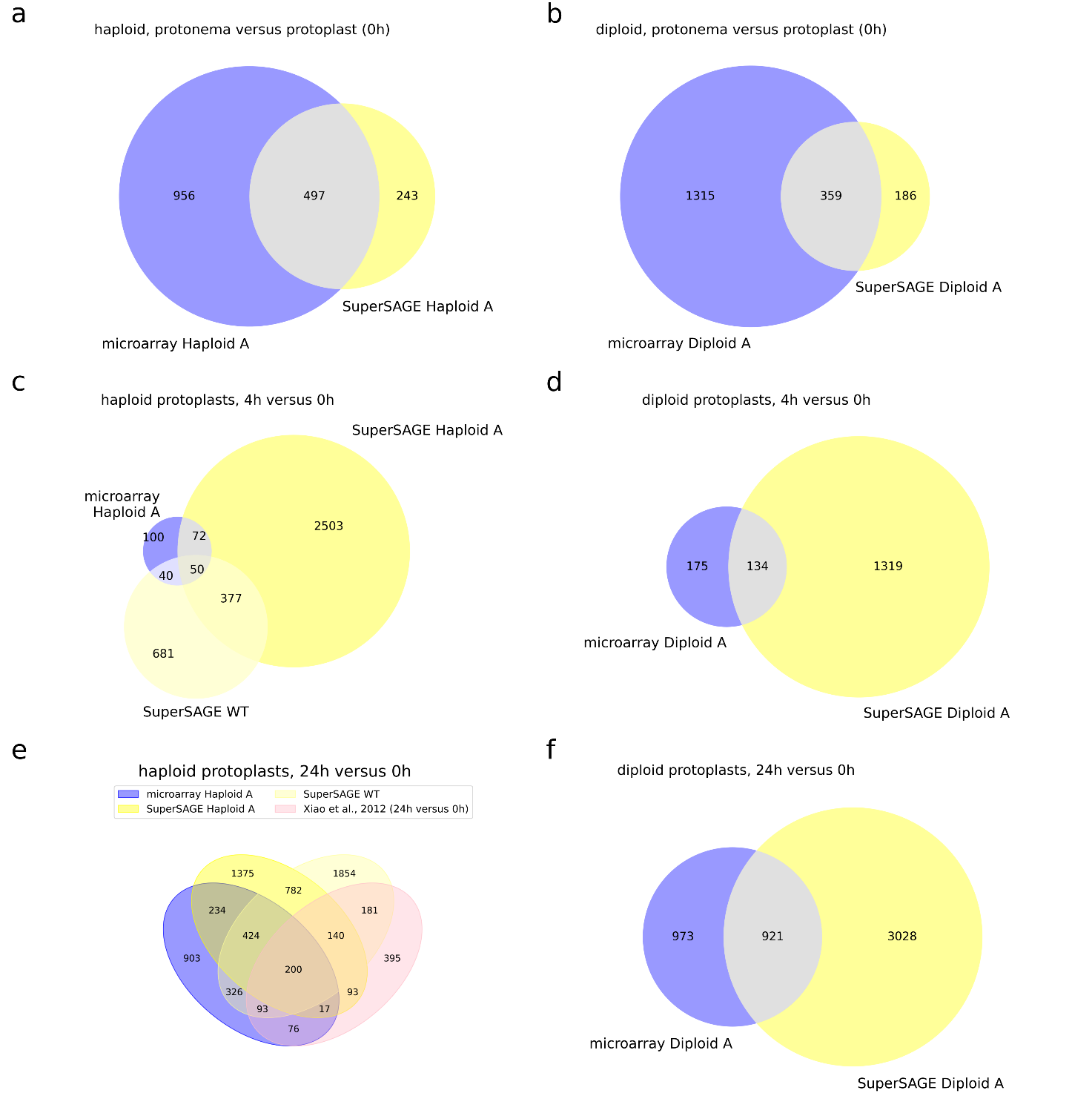
**

**Supplementary Table T7** Number of differentially expressed genes (DEGs) between diploid and haploid lines in freshly isolated protoplasts (0h), in protoplasts at 4h and 24h after transfection as well as in protonema cells (PN). Comparisons were performed on microarray and SuperSAGE data from different lines. DEGs from the microarray experiment were determined with the Expressionist Analyst Pro software and filtered for a |log2 fold change| > 1 and p < 0.001. The SuperSAGE data analysis was performed with GFOLD and DEGs were filtered for a GFOLD(0.01) value of < -1 or > 1.

| **Comparison** | **Experiment** | **0 h** | **4h** | **24h** | **PN** |
| --- | --- | --- | --- | --- | --- |
| **Diploid A vs. Haploid A** | Microarray | 36 | 88 | 43 | 67 |
| **Diploid A, Diploid B vs.**  **WT, Haploid A** | Microarray | 9 | 20 | 11 | 55 |
| **Diploid A vs. WT** | SuperSAGE | 1723 | 977 | 870 | - |
| **Diploid A vs. Haploid A** | SuperSAGE | 416 | 2317 | 3001 | 0 |


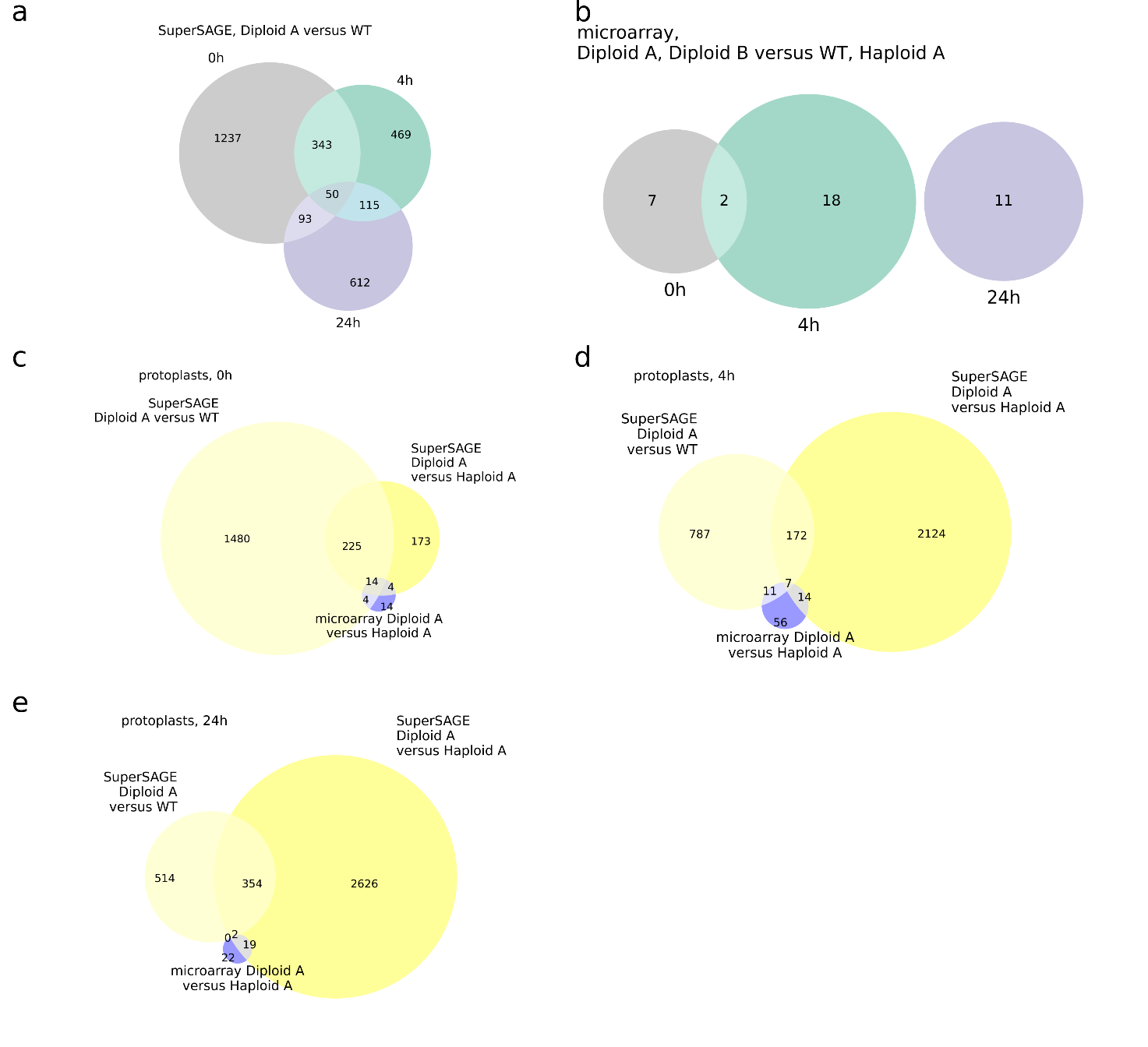
**Supplementary Figure S4** Overlap of differentially expressed genes (DEGs) identified from pairwise comparison between haploid and diploid protoplast cells. Shown is the overlap between DEGs at different protoplast ages (grey: freshly isolated protoplasts (0h), green: protoplasts at 4h after transfection, purple: protoplasts at 24h after transfection) from Diploid A versus WT using SuperSAGE libraries (a) and from Diploid A and Diploid B versus WT and Haploid A using microarray libraries (b). Further, the overlap of DEGs from microarray (blue) and SuperSAGE data (yellow) at 0h (c), 4h after transfection (d) and 24h after transfection (e) is illustrated. DEGs from the microarray experiment were determined with the Expressionist Analyst Pro software and were filtered for |log2 fold change| > 1 and p < 0.001. The SuperSAGE data analysis was performed with GFOLD and DEGs were filtered for a GFOLD(0.01) value of < -1 or > 1.

Supplementary Figure S5 Comparison of variance between samples used for SuperSAGE library construction via Principle Component Analysis (PCA). Similarity of haploid and diploid lines and tissue (including protoplast age) is shown. Tissue type and protoplast age are the main factors of the variance encountered.


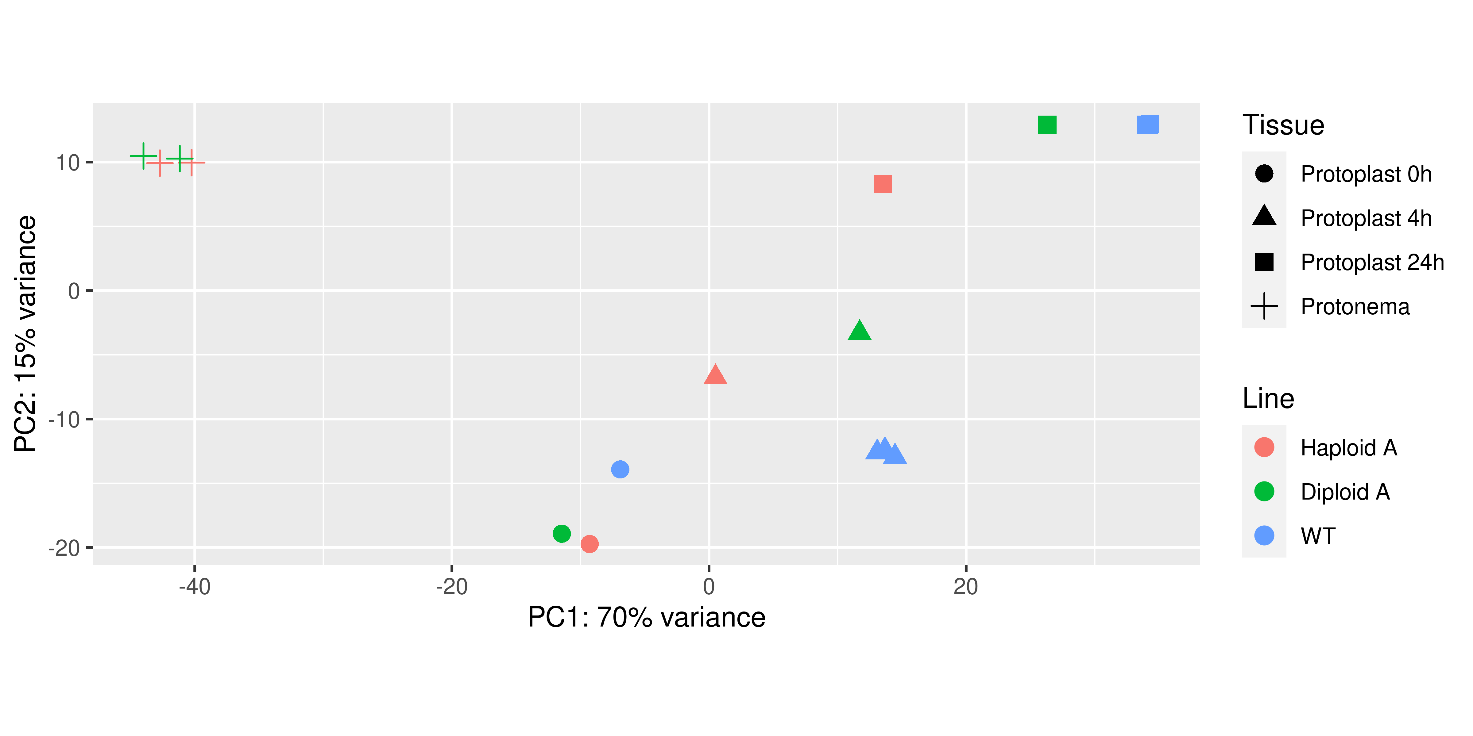


Supplementary Table T8 Overview of the differentially expressed genes (DEGs) with the highest fold changes in gene expression between the diploid line Diploid A and the haploid WT. Listed are the 10 DEGs with the strongest upregulation as well as the 10 DEGs with the strongest downregulation. Statistical analysis was performed with DESeq2 using a two-factor design to test for ploidy-dependent expression factoring in the differences derived from different developmental stages. Only SuperSAGE libraries of protoplast samples were included in the analysis (Supplementary Table T3). Expression fold changes are given for diploid cells in comparison to haploid cells. The annotation is based on Phytozome (v12.1.5; Goodstein et al. 2012).

| **V33 ID** | **Description** | **Log2 fold change** | **Adjusted p value** |
| --- | --- | --- | --- |
| **Pp3c23_940** | PF07470 – Glycosyl Hydrolase Family 88 (Glyco_hydro_88) (1 of 5) | 3.50 | 1.64E-15 |
| **Pp3c22_10130** | K05605 – 3-hydroxyisobutyryl-CoA hydrolase (HIBCH) (1 of 4) | 3.44 | 2.66E-17 |
| **Pp3c4_17790** | not annotated | 3.16 | 1.04E-17 |
| **Pp3c4_25770** | PTHR28039:SF4 – CHALCONE–FLAVONONE ISOMERASE 3-RELATED (1 of 2) | 3.08 | 1.32E-14 |
| **Pp3c2_21940** | not annotated | 3.02 | 1.65E-10 |
| **Pp3c1_18940** | K10775 – phenylalanine ammonia-lyase (E4.3.1.24) (1 of 12) | 2.61 | 2.76E-11 |
| **Pp3c4_24450** | not annotated | 2.55 | 2.54E-21 |
| **Pp3c2_30370** | not annotated | 2.53 | 8.83E-10 |
| **Pp3c25_14520** | not annotated | 2.52 | 1.57E-20 |
| **Pp3c6_6560** | PTHR21495:SF39 – DIRIGENT PROTEIN 16-RELATED (1 of 8) | 2.51 | 6.04E-08 |
| **Pp3c4_20120** | 1.1.3.9 – Galactose oxidase / Beta-galactose oxidase (1 of 36) | -2.17 | 2.56E-05 |
| **Pp3c3_16280** | PTHR31867:SF30 – EXPANSIN-A5 (1 of 29) | -2.18 | 5.18E-05 |
| **Pp3c24_12370** | PTHR11069 – GLUCOSYLCERAMIDASE (1 of 1) | -2.20 | 2.08E-06 |
| **Pp3c21_14285** | not annotated | -2.24 | 1.21E-04 |
| **Pp3c21_14239** | not annotated | -2.28 | 8.23E-05 |
| **Pp3c12_15190** | PTHR31989:SF39 – NAC DOMAIN-CONTAINING PROTEIN 12 (1 of 10) | -2.31 | 2.50E-08 |
| **Pp3c13_12570** | PTHR24067:SF41 – AKT-INTERACTING PROTEIN (1 of 1) | -2.39 | 1.14E-06 |
| **Pp3c21_14233** | KOG0472 – Leucine-rich repeat protein (1 of 34) | -2.44 | 1.75E-05 |
| **Pp3c18_19690** | PTHR31867:SF30 – EXPANSIN-A5 (1 of 29) | -2.63 | 1.51E-11 |
| **Pp3c4_19000** | K02977 – small subunit ribosomal protein S27Ae (RP-S27Ae, RPS27A) (1 of 4) | -3.25 | 3.96E-44 |

Supplementary Table T9 Overview of the differentially expressed genes (DEGs) with the highest fold changes in gene expression between a diploid line (Diploid A) and two haploid lines (WT, Haploid A). Listed are the 10 DEGs with the strongest upregulation as well as the 10 DEGs with the strongest downregulation. Statistical analysis was performed with DESeq2 using a two-factor design to test for ploidy-dependent expression factoring in the differences derived from different developmental stages. All 17 SuperSAGE libraries of protoplast and protonema samples (Supplementary Table T3) were included in the analysis. Expression fold changes are given for diploid cells in comparison to haploid cells. The annotation is based on Phytozome (v12.1.5; Goodstein et al., 2012).

| **V33 ID** | **Description** | **Log2 fold change** | **Adjusted p value** |
| --- | --- | --- | --- |
| **Pp3c20_15490** | PTHR11360 - MONOCARBOXYLATE TRANSPORTER (1 of 1) | 1.56 | 2.21E-04 |
| **Pp3c4_17790** | not annotated | 1.53 | 2.20E-03 |
| **Pp3c23_940** | PF07470 - Glycosyl Hydrolase Family 88 (Glyco_hydro_88) (1 of 5) | 1.52 | 2.90E-03 |
| **Pp3c3_810** | PTHR11514//PTHR11514:SF57 - MYC // SUBFAMILY NOT NAMED (1 of 5) | 1.52 | 9.75E-06 |
| **Pp3c13_18730** | not annotated | 1.51 | 9.51E-05 |
| **Pp3c7_20720** | PF02743//PF13519 - Cache domain (Cache_1) // von Willebrand factor type A domain (VWA_2) (1 of 1) | 1.47 | 2.00E-03 |
| **Pp3c4_20080** | K01087 - trehalose 6-phosphate phosphatase (otsB) (1 of 6) | 1.47 | 1.09E-04 |
| **Pp3c11_2950** | K09753 - cinnamoyl-CoA reductase (CCR) (1 of 2) | 1.44 | 2.05E-03 |
| **Pp3c15_4030** | K12640 - brassinosteroid-6-oxidase 2 (CYP85A2, BR6OX2) (1 of 1) | 1.44 | 2.47E-03 |
| **Pp3c4_2660** | PF00847 - AP2 domain (AP2) (1 of 150) | 1.43 | 3.63E-05 |
| **Pp3c4_30220** | PTHR19139:SF90 - AQUAPORIN PIP1-5-RELATED (1 of 3) | -1.53 | 2.43E-03 |
| **Pp3c1_42230** | not annotated | -1.55 | 2.33E-03 |
| **Pp3c1_42250** | not annotated | -1.55 | 2.33E-03 |
| **Pp3c1_42420** | not annotated | -1.55 | 2.33E-03 |
| **Pp3s350_10** | not annotated | -1.55 | 2.33E-03 |
| **Pp3c2_8570** | not annotated | -1.58 | 2.29E-04 |
| **Pp3c4_20120** | 1.1.3.9 - Galactose oxidase / Beta-galactose oxidase (1 of 36) | -1.69 | 2.37E-04 |
| **Pp3c20_8180** | K14500 - BR-signalling kinase (BSK) (1 of 4) | -1.71 | 9.51E-05 |
| **Pp3c17_19460** | PF04885 - Stigma-specific protein, Stig1 (Stig1) (1 of 7) | -1.85 | 4.87E-05 |
| **Pp3c3_16280** | PTHR31867:SF30 - EXPANSIN-A5 (1 of 29) | -2.28 | 2.04E-08 |

Supplementary Table T10 Differentially expressed genes (DEGs) between diploid and haploid cells involved in developmental or growth processes. From the two-factor analyses DEGs with a |log2 fold change| > 1.5 were selected if they had a biological process GO term assigned considering development process (GO:0032502), growth (GO:0040007) or any of their child terms. Only these GO terms are listed in the table. DEGs in the first two-factor analysis (I) were computed from Diploid A versus WT and Haploid A using protoplast and protonema samples whereas DEGs in the second two-factor analysis (II) were computed from Diploid A versus WT using only protoplast samples. The functional annotation is based on Phytozome (v12.1.5; Goodstein et al., 2012) and the GO terms are from the PpGML DB (Fernandez‐Pozo et al., 2020).

| **Gene ID** | **Description** | **GO term biological process** | **Log2 fold change** | **Adjusted p value** |
| --- | --- | --- | --- | --- |
| **Pp3c9_22230** | PTHR10593//PTHR10593:SF27 - SERINE/THREONINE-PROTEIN KINASE RIO // SUBFAMILY NOT NAMED (1 of 5) | pollen tube growth | 1.88 (in II) | 2.37E-12 (in II) |
| **Pp3c1_10860** | 2.7.11.25 - Mitogen-activated protein kinase kinase kinase / MLTK (1 of 32) | anatomical structure development | 1.85 (in II) | 2.07E-06 (in II) |
| **Pp3c12_20650** | 1.3.1.33 - Protochlorophyllide reductase / Protochlorophyllide oxidoreductase (1 of 2) | leaf morphogenesis, cell differentiation | 1.82 (in II) | 7.75E-09 (in II) |
| **Pp3c15_4030** | K12640 - brassinosteroid-6-oxidase 2 (CYP85A2, BR6OX2) (1 of 1) | skotomorphogenesis | 1.44 (in I)  1.81 (in II) | 2.47E-3 (in I)  3.41E-4 (in II) |
| **Pp3c22_2330** | PTHR13398 - GDP-FUCOSE PROTEIN O-FUCOSYLTRANSFERASE 2 (1 of 5) | leaf morphogenesis, cell differentiation | 1.57 (in II) | 6.47E-3 (in II) |
| **Pp3c13_1640** | K01728 - pectate lyase (pel) (1 of 23) | multidimensional cell growth, root hair elongation, cell tip growth, regulation of meristem growth, pattern specification process | 1.13 (in I)  1.56 (in II) | 1.48E-2 (in I)  3.68E-3 (in II) |
| **Pp3c27_7000** | 1.5.1.9 - Saccharopine dehydrogenase (NAD(+), L-glutamate-forming) / Saccharopin dehydrogenase (1 of 1) | vegetative to reproductive phase transition of meristem | -1.54 (in II) | 3.53E-8 (in II) |
| **Pp3c5_21920** | K03065 - 26S proteasome regulatory subunit T5 (PSMC3, RPT5) (1 of 2) | pollen development, embryo sac egg cell differentiation | -1.72 (in II) | 2.50E-08 (in II) |
| **Pp3c10_19880** | PTHR33219:SF1 - COFACTOR ASSEMBLY, COMPLEX C (B6F) (1 of 1) | leaf morphogenesis, cell differentiation | -1.73 (in II) | 1.45E-3 (in II) |

## Supplementary Figure S6 XRCC4 expression in the Reute and Gransden Physcomitrella ecotypes at different developmental stages. Expression values were obtained from the PEATmoss website and only the four datasets containing values for the gene expression in the sporophyte were selected. Gene expression in the datasets CombiMatrix Developmental stages (a), NimbleGen Developmental and Mycorrhiza (b), NimbleGen Ortiz-Ramírez et al., 2016 (c) and RNAseq developmental stages (d) is shown. In the NimbleGen Developmental and Mycorrhiza dataset mycorrhiza exudate and heat treated samples were not considered. Hatched bars represent expression values from the Reute ecotype. Abbreviations: BlqA = BCDA (ammonium) liquid, Bsl = BCD solid, BslA = BCDA (ammonium) solid, Klq = Knop liquid, KlqA = Knop liquid ammonium, Ksl = Knop solid. Colours of source material: Spores in blue, protonema in orange, gametophores in purple, sporophyte in pink, protoplast in turquoise, archegonia in brown, rhizoids in grey, chloronema in red, caulonema in green, leaflets in olive.


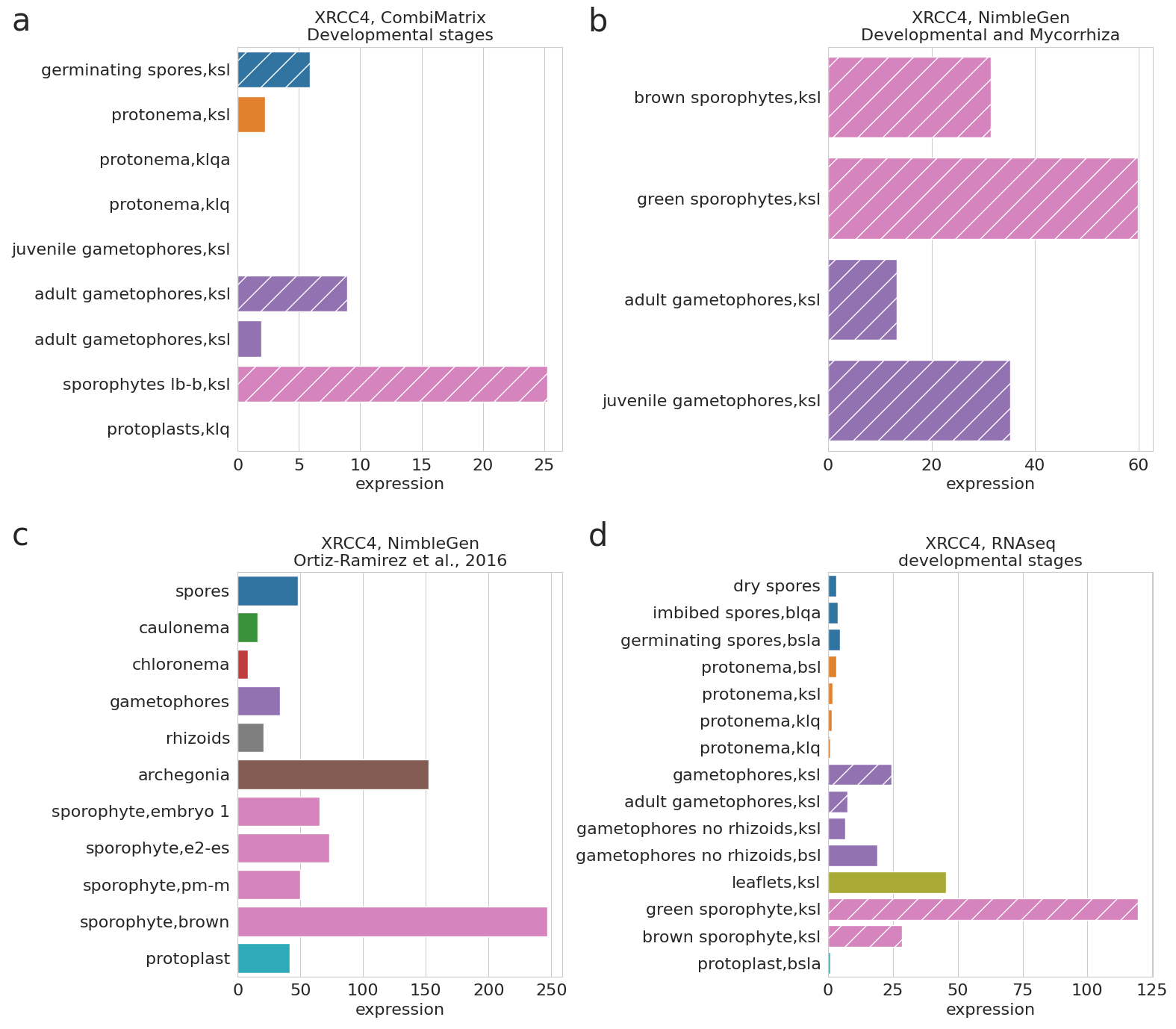


## Supplementary Figure S7 Cyclin D2 expression in the Reute and Gransden Physcomitrella ecotypes at different developmental stages. Expression values were obtained from the PEATmoss website and only the four datasets containing values for the gene expression in the sporophyte were selected. The gene expression in the datasets CombiMatrix Developmental stages (a), NimbleGen Developmental and Mycorrhiza (b), NimbleGen Ortiz-Ramírez et al., 2016 (c) and RNAseq developmental stages (d) is shown. In the NimbleGen Developmental and Mycorrhiza dataset mycorrhiza exudate and heat treated samples were not considered. Hatched bars represent expression values from the Reute ecotype. Abbreviations: BlqA = BCDA (ammonium) liquid, Bsl = BCD solid, BslA = BCDA (ammonium) solid, Klq = Knop liquid, KlqA = Knop liquid ammonium, Ksl = Knop solid. Colours of source material: Spores in blue, protonema in orange, gametophores in purple, sporophyte in pink, protoplast in turquoise, archegonia in brown, rhizoids in grey, chloronema in red, caulonema in green, leaflets in olive.


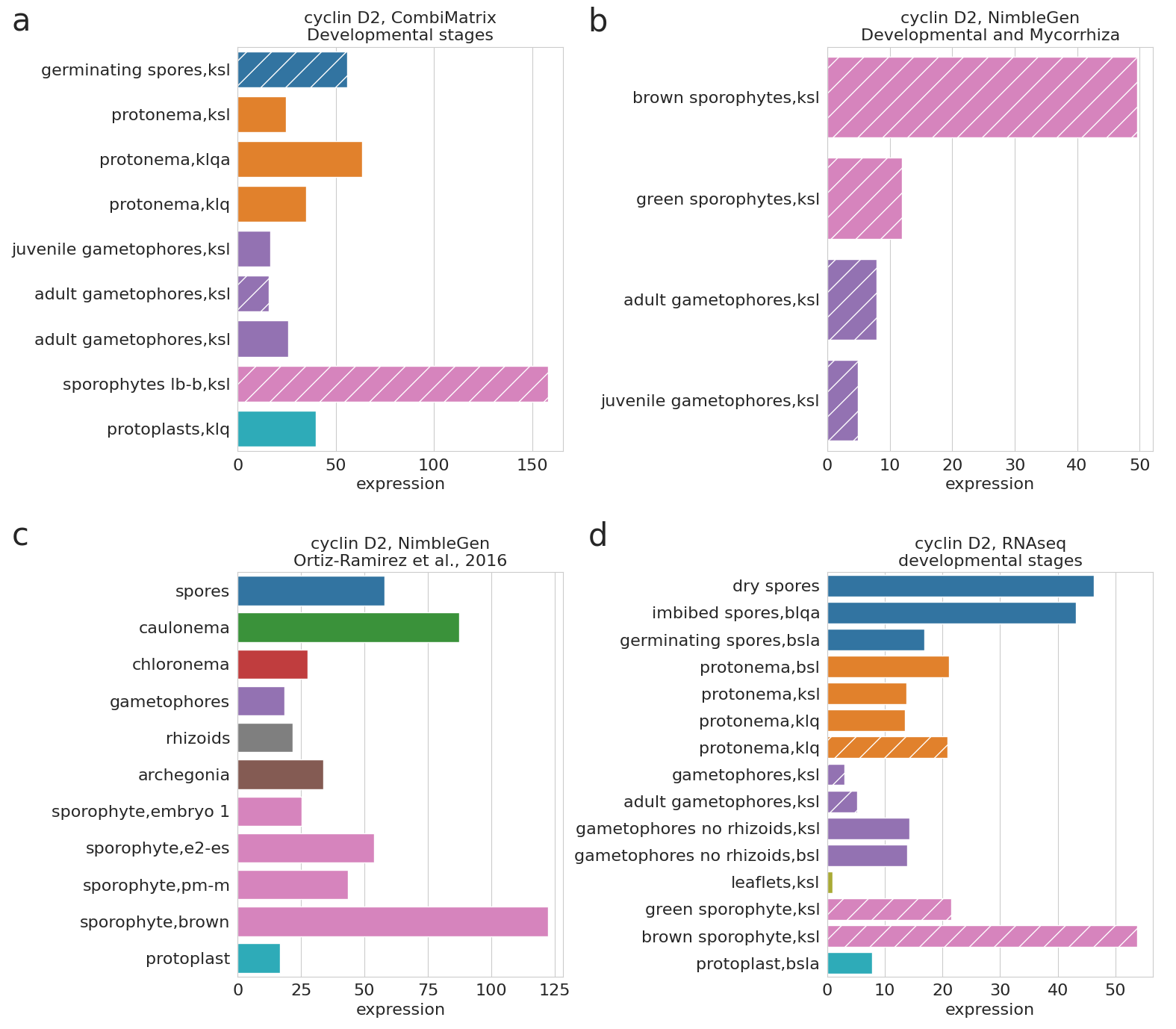


## Supplementary Figure S8 CENPE expression in the Reute and Gransden Physcomitrella ecotypes at different developmental stages. Expression values were obtained from the PEATmoss website and only the four datasets containing values for the gene expression in the sporophyte were selected. The gene expression in the datasets CombiMatrix Developmental stages (a), NimbleGen Developmental and Mycorrhiza (b), NimbleGen Ortiz-Ramírez et al., 2016 (c) and RNAseq developmental stages (d) is shown. In the NimbleGen Developmental and Mycorrhiza dataset mycorrhiza exudate and heat treated samples were not considered. Hatched bars represent expression values from the Reute ecotype Abbreviations: BlqA = BCDA (ammonium) liquid, Bsl = BCD solid, BslA = BCDA (ammonium) solid, Klq = Knop liquid, KlqA = Knop liquid ammonium, Ksl = Knop solid. Colours of source material: Spores in blue, protonema in orange, gametophores in purple, sporophyte in pink, protoplast in turquoise, archegonia in brown, rhizoids in grey, chloronema in red, caulonema in green, leaflets in olive.


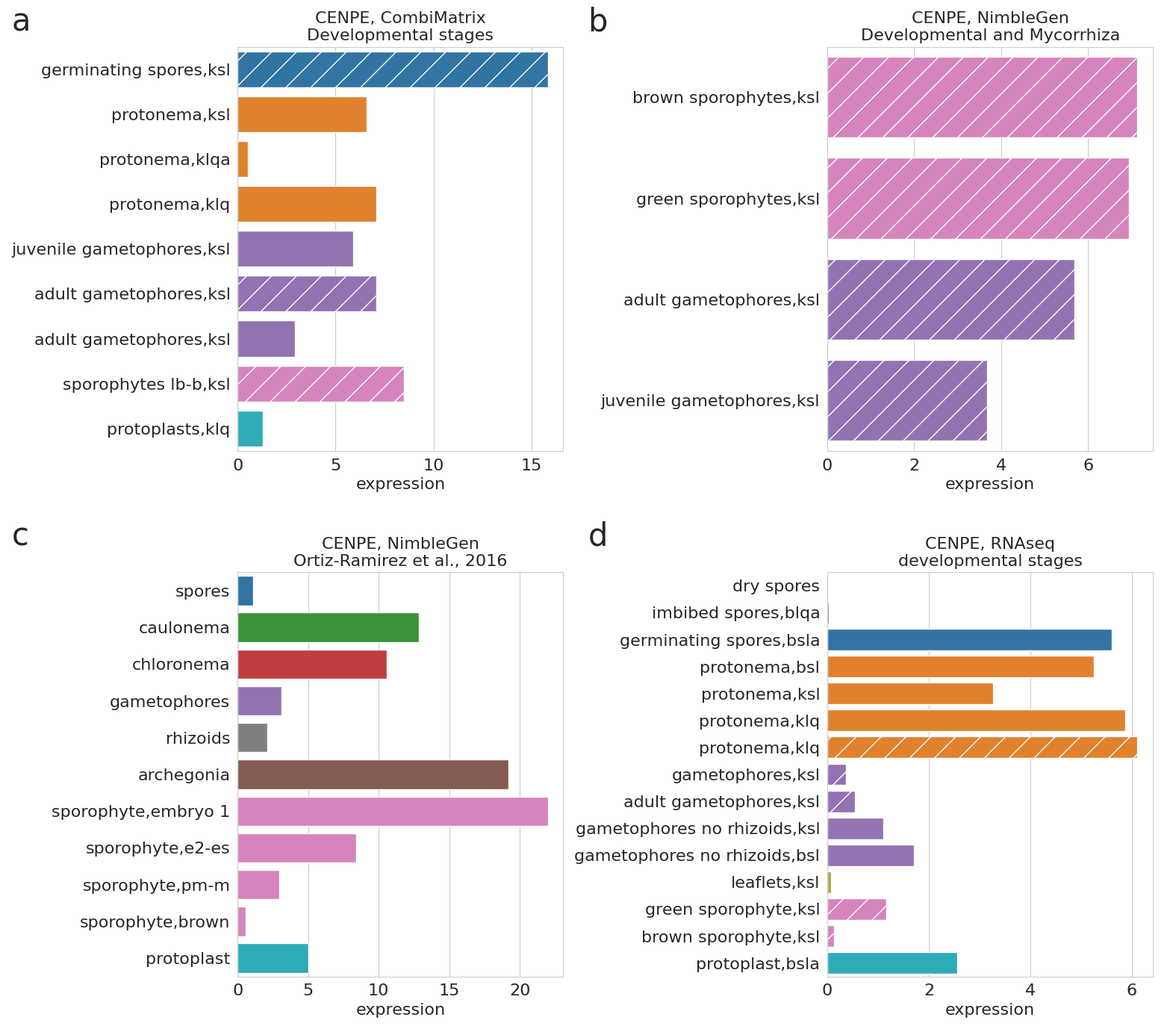


## Supplementary Figure S9 H3K4 expression in the Reute and Gransden Physcomitrella ecotypes at different developmental stages. Expression values were obtained from the PEATmoss website and only the four datasets containing values for the gene expression in the sporophyte were selected. The gene expression in the datasets CombiMatrix Developmental stages (a), NimbleGen Developmental and Mycorrhiza (b), NimbleGen Ortiz-Ramírez et al., 2016 (c) and RNAseq developmental stages (d) is shown. In the NimbleGen Developmental and Mycorrhiza dataset mycorrhiza exudate and heat treated samples were not considered. Hatched bars represent expression values from the Reute ecotype Abbreviations: BlqA = BCDA (ammonium) liquid, Bsl = BCD solid, BslA = BCDA (ammonium) solid, Klq = Knop liquid, KlqA = Knop liquid ammonium, Ksl = Knop solid. Colours of source material: Spores in blue, protonema in orange, gametophores in purple, sporophyte in pink, protoplast in turquoise, archegonia in brown, rhizoids in grey, chloronema in red, caulonema in green, leaflets in olive.


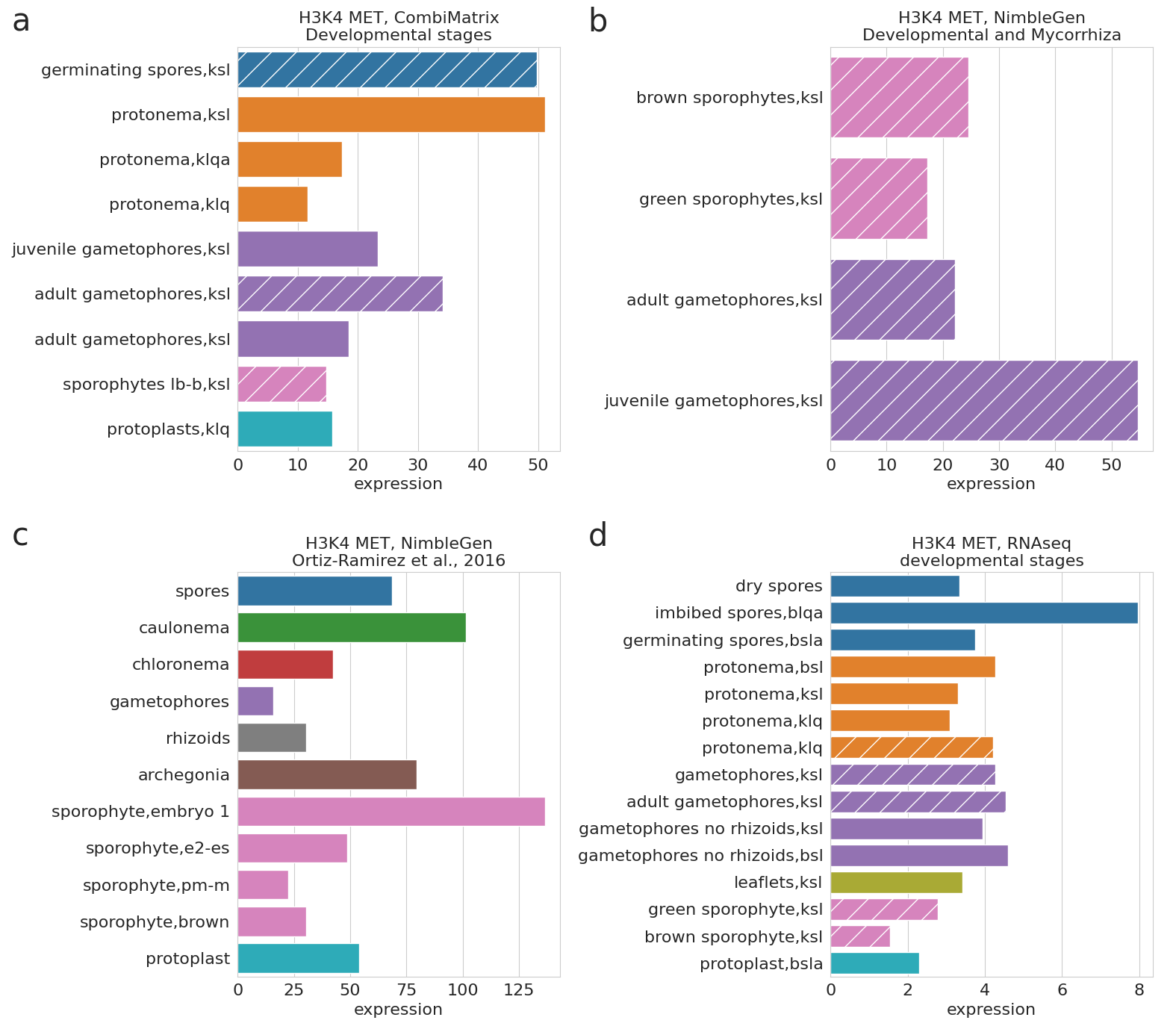

Supplement: Supplementary file 1 — Supplementary file1 (DOCX 1408 KB) [file 299_2021_2794_MOESM1_ESM.docx]
